# Supplementary material for: Cross-ethnicity/race generalization failure of behavioral prediction from resting-state functional connectivity
Source: Sci Adv. 2022 Mar 16;8(11):eabj1812. doi: 10.1126/sciadv.abj1812 (PMC8926333; doi:10.1126/sciadv.abj1812)
Supplement: 20220318-1 [file sciadv.abj1812.v1.pdf]

NEUROSCIENCE

Copyright © 2022 The Authors, some rights reserved; exclusive licensee American Association for the Advancement of Science. No claim to original U.S. Government Works. Distributed under a Creative Commons Attribution License 4.0 (CC BY).

Cross-ethnicity/race generalization failure of behavioral prediction from resting-state functional connectivity

Jingwei Li[1,2,3]*, Danilo Bzdok[4,5], Jianzhong Chen[3], Angela Tam[3], Leon Qi Rong Ooi[3], Avram J. Holmes[6,7], Tian Ge[7,8,9], Kaustubh R. Patil[1,2], Mbemba Jabbi[10,11,12,13], Simon B. Eickhoff[1,2], B. T. Thomas Yeo[3,14]*†, Sarah Genon[1,2]*†

Algorithmic biases that favor majority populations pose a key challenge to the application of machine learning for precision medicine. Here, we assessed such bias in prediction models of behavioral phenotypes from brain functional magnetic resonance imaging. We examined the prediction bias using two independent datasets (preadolescent versus adult) of mixed ethnic/racial composition. When predictive models were trained on data dominated by white Americans (WA), out-of-sample prediction errors were generally higher for African Americans (AA) than for WA. This bias toward WA corresponds to more WA-like brain-behavior association patterns learned by the models. When models were trained on AA only, compared to training only on WA or an equal number of AA and WA participants, AA prediction accuracy improved but stayed below that for WA. Overall, the results point to the need for caution and further research regarding the application of current brain-behavior prediction models in minority populations.

INTRODUCTION

Predictive models, capitalizing on machine learning algorithms, are expected to play an important role in precision medicine (*1*–*3*). Benefits of their application have already been demonstrated for disease diagnostics, drug sensitivity prediction, and biomarker discovery in multiple studies. For example, a machine learning model was designed to identify drug-sensitive molecular markers for acute myeloid leukemia (*4*), and deep learning has been used to detect diabetic retinopathy (*5*). Furthermore, several machine learning–based cardiometabolic biomarkers have been shown to be better at predicting diabetes than traditional clinical biomarkers (*6*). Thus, the future of precision medicine, from disease prevention to diagnostics and treatment, will likely be shaped by the deployment of machine learning–based models for individual prediction. From the neuroscience perspective, applying machine learning–based models on neuroimaging data and cognitive/psychometric phenotypes can potentially facilitate better diagnostics and treatment of mental disorders, allowing for patient clustering and outcome prediction, while improving our understanding of individual differences in brain and behavior.

However, despite the promise of predictive modeling approaches, concerns have been raised that machine learning algorithms and related data mining techniques might not alleviate but rather increase bias and hence unfairness against specific subpopulations (*7*–*9*). For instance, many commercial gender classification systems exhibited higher prediction error rate for darker-skinned females than for lighter-skinned males (*10*). In more sensitive applications, model unfairness could create marked consequences for specific groups, e.g., higher predicted crime rate than actual rate or lower expected health care investment than necessary. Several reasons could jointly lead to such unfairness (*11*): (i) an insufficient representation of minority groups in the data on which the model is built, (ii) the predictive models fitting more to majority groups during training optimization, and (iii) historical societal bias in the data (i.e., machine learning models may learn from previous discrimination/bias caused by societal reasons, e.g., gender inequality in indices of professional success).

In the face of this complex, multicausal issue, research on algorithmic fairness has rapidly evolved over the recent years. The main and ultimate objective is to align algorithmic systems with broader societal goals across social, ethical, and legal contexts. However, under different application scenarios, the concept of "fairness" pertains to different aspects. This heterogeneity leads to diverse strategies to promote fairness in different fields of algorithm application (*12*). For example, independence of a sensitive attribute (such as demographic disparity) on model predictions (*13*) might be considered in a school admission process to ensure that students with different socioeconomic backgrounds receive the same education opportunity. If a model that systematically underestimates outcomes for an unprivileged group could be harmful (e.g., lower true-positive rate to detect a disease in a minority ethnic group), equalized sensitivity should be implemented to correct this bias (*14*, *15*). Similar strategies for different fairness objectives include equalized odds (*14*), calibration-based criteria (*16*), and net compensation (*17*). Given ongoing debates, not only in science but also in humanities, there might not be a solution that would satisfy all

[1]Institute of Neuroscience and Medicine, Brain and Behavior (INM-7), Research Center Jülich, Jülich, Germany. [2]Institute for Systems Neuroscience, Medical Faculty, Heinrich-Heine University Düsseldorf, Düsseldorf, Germany. [3]Department of Electrical and Computer Engineering, Centre for Sleep and Cognition, Centre for Translational Magnetic Resonance Research, and N.1 Institute for Health and Institute for Digital Medicine, National University of Singapore, Singapore, Singapore. [4]Department of Biomedical Engineering, Montreal Neurological Institute (MNI), McConnell Brain Imaging Institute (BIC), McGill University, Montreal, QC, Canada. [5]Mila-Quebec Artificial Intelligence Institute, Montreal, QC, Canada. [6]Departments of Psychology and Psychiatry, Yale University, New Haven, CT, USA. [7]Psychiatric and Neurodevelopmental Genetics Unit, Center for Genomic Medicine, Massachusetts General Hospital, Boston, MA, USA. [8]Stanley Center for Psychiatric Research, Broad Institute of MIT and Harvard, Cambridge, MA, USA. [9]Department of Psychiatry, Massachusetts General Hospital, Harvard Medical School, Boston, MA, USA. [10]Department of Psychiatry, Dell Medical School, University of Texas at Austin, Austin, TX, USA. [11]The Mulva Clinic for Neurosciences, Dell Medical School, University of Texas at Austin, Austin, TX, USA. [12]Institute of Neuroscience, University of Texas at Austin, Austin, TX, USA. [13]Department of Psychology, University of Texas at Austin, Austin, TX, USA. [14]Integrative Sciences and Engineering Programme (ISEP), National University of Singapore, Singapore, Singapore.
*Corresponding author. Email: jingweili.sjtu.nus@gmail.com (J.L.); s.genon@fz-juelich.de (S.G.); thomas.yeo@nus.edu.sg (B.T.T.Y.)
†These authors contributed equally to this work.

conceivable, at times mutually incompatible, aspects of fairness within every usage context (*15*). It has also been raised that simply considering algorithmic fairness per se is not enough. Rather, model developers also need to collaborate with the stakeholders, such as patient advocacy groups for health care applications. Such collaborations contribute, for example, to identify possible bias in data collection (*12*).

In neuroscience, one major line of research aims to predict behavioral phenotype from brain data, in particular using resting-state/task/naturalistic functional magnetic resonance imaging (fMRI) (*18*–*22*). In that context, the prevalent predictive models have been mostly trained on datasets mixing multiple ethnicities/races. However, little or no attention has been given to the cross-ethnicity/race fairness of these predictive models while the datasets were usually dominated by participants with European ancestry and/or white ethnic/racial background (*23*, *24*). Given the recent reports of bias toward specific populations in machine learning, the question can be raised whether brain-based prediction models can perform as well for minority ethnic/racial groups as for the majority population. Although emerging efforts have been made to build machine learning models on specific non-white/European populations, e.g., Chinese population (*25*), comparisons of prediction across ethnicities/races are still lacking. A wide range of socioeconomical, cultural, educational, and biological variables can individually, or in interaction, complicate the identification of a common pattern of brain-behavior associations across different populations (*26*–*28*).

The assessment and characterization of unfair discrepancies in the prediction of behavioral phenotypes should be of particular importance to the field. While still in its inception, brain-based predictive approaches of behavioral phenotypes have the potential to contribute to our understanding of brain-behavior relationships in humans. These approaches should ultimately lead to the development of new clinical biomarkers and the prediction of individual treatment trajectories and hence spur progresses in precision medicine. In this context, unfairness issues in brain-based prediction of phenotypic variability could increase the impacts of structural racism and negatively affect patient care.

In the present study, we investigated how predictive models of behavioral phenotypes from resting-state functional connectivity (RSFC) data generalized across populations. RSFC-based models were used in this study for both conceptual and practical reasons. First, from a conceptual standpoint, behavioral functions are supported by distributed functional networks. Accordingly, most brain-based prediction studies have been based on, or at least included, RSFC (rather than brain structural features) as brain predicting features. Thus, by focusing on RSFC, the current study reflects the contemporary scope of the brain-based prediction field. Second, compared to task-based fMRI, using resting-state fMRI (rs-fMRI) data has two advantages: (i) From a clinical perspective, resting-state data are more readily collected, especially in children, patients with cognitive impairment, and in elderly people. (ii) From a practical standpoint, the current study requires optimizing (i.e., maximizing) the sample size (in particular for minorities) and hence benefitted from the greater data availability for resting-state data as compared to task fMRI data. More specifically, we examined whether white Americans (WA) and African Americans (AA) enjoyed similar prediction performance when the predictive models were trained on state-of-the-art large-scale datasets. The terminology of ethnicities/races in this study followed the National Institutes of Health definitions of racial and ethnic categories (https://grants.nih.gov/grants/guide/notice-files/not-od-15-089.html).

We used two publicly available large datasets of the U.S. population to ensure that our findings were not specific to a specific age range, scanning process (single versus multisite), and preprocessing strategy. Likewise, by focusing on U.S. data, we avoided influences arising from comparisons across different cultural societies, including school and health care systems, food preferences, and other factors, although we note that these may also exist within the United States. Behavioral predictive models were built using the kernel ridge regression method. We first compared the prediction accuracy, measured by predictive coefficient of determination (COD), between phenotypically matched WA and AA, when models were trained on the entire sample (mixing all ethnic/racial groups, not only AA and WA). The objective of this training on diverse populations was to mimic the dominating approach currently taken in the field for building predictive models.

To evaluate model accuracy in regression problems in a fairness framework, previous studies have used COD (*17*, *29*), mean squared error (MSE) (*30*), or the loss of their optimization function as accuracy metrics (*31*). Using MSE and loss of cost function in this study would have resulted in discrepant scales for different behavioral phenotypes. Noting that, mathematically, predictive COD and MSE are inversely related (see the last section of Results for further information), we here opted for the former. Nevertheless, because correlation has been and is still often used for evaluating model performance within the brain-based prediction field, we repeated all analyses using Pearson's correlation as an accuracy metric.

Because an important source of the unfairness in machine learning algorithms may arise from insufficient representation in the training set for minority populations, we further investigated the effects of training population on the differences in test accuracies between AA and WA. Specifically, we compared predictive models trained on AA only, on WA only, or on a perfectly balanced (half AA–half WA) set. Last, we also investigated potential mechanisms underlying different model performance for AA and WA. It can hence be assumed that good performance of a predictive model in one population goes hand in hand with the learning of a valid representation of the association between features and target variables from the model in that specific population. Thus, we here also examined the relationship between the prediction accuracy in a certain ethnic/racial group and how well the predictive model captured the brain-behavior association patterns in that group.

RESULTS

Full-dataset model (kernel ridge regression) yielded higher prediction error in AA than in WA

We used two large-scale datasets containing both neuroimaging and behavioral data from the U.S. population: the Human Connectome Project (HCP; $N = 948$; age, 22 to 37 years) and the Adolescent Brain Cognitive Development (ABCD; $N = 5351$; age, 9 to 11 years). Each dataset included various reported ethnicities/races, but they were both heavily dominated in numbers by WA (Fig. 1, A and B). Ethnic/racial groups were categorized on the basis of self-reported data. Kernel ridge regression models were trained on the entire datasets including all ethnic/racial groups to predict each behavioral measure from RSFC, with accuracy being assessed by state-of-the-art cross-validation procedures. While determining an optimal algorithm for

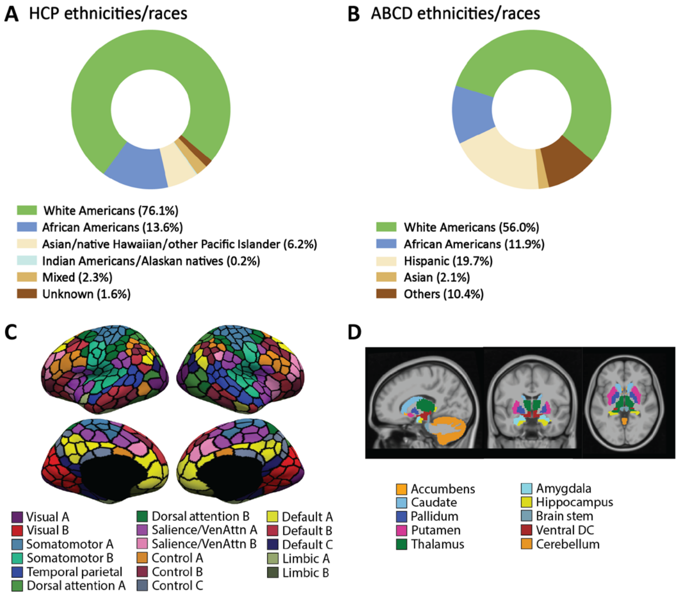

Fig. 1. Ethnic/racial composition in our datasets and the brain atlases used for RSFC calculation. Subpopulation composition of (**A**) HCP and (**B**) ABCD and functional connectivity ROIs. Note that the naming of ethnic/racial categories in (B) followed the definition given by the ABCD consortium (*68*), which was slightly different from the National Institutes of Health definition. (**C**) The 400-area cortical parcellation derived by Schaefer *et al.* (*56*). Parcel colors correspond to 17 large-scale networks (*95*). (**D**) Nineteen subcortical ROIs from Deskian/Killiany atlas (*89*).

MRI-based behavioral prediction was still an undergoing research topic, kernel ridge regression was selected as the main prediction algorithm in this work because it has been shown to be a fast and effective method for predicting phenotypes from fMRI (*32*). However, our main analyses were also repeated using linear ridge regression (see below). Confounding variables were regressed from both RSFC and behavioral scores (see Materials and Methods). First, as a main analysis, predictive model performance was compared between matched test sets of AA and WA using predictive COD [as recommended in (*33*)]. The purpose of matching between AA and WA was to exclude potential accuracy differences that would be attributable to differences in basic demographic/anthropometric/data quality variables (such as age, gender, and in-scanner movements) between the two groups rather than to ethnicity/race.

HCP dataset

For the HCP dataset, first, AA participants were randomly split into 10 folds (Fig. 2A). Within each fold of AA, Hungarian matching was performed to assign a WA participant (without repetition) to each individual AA so that the differences between the matched AA and WA in behavioral scores and confounding variables were minimized. Predictive models were trained on nine folds and tested on the remaining fold in a cross-validated manner. Following the dominating approach to build predictive models in this field, which usually mixed all ethnic/racial groups in a dataset, the matched AA and WA from training folds were grouped with 90% of randomly selected participants from other ethnic/racial groups and the unmatched WA and AA. The whole procedure was repeated randomly 40 times to ensure that the results were not driven by the initial split of folds. For 51 of the total 58 behavioral measures, AA and WA could be matched (fig. S1). See Materials and Methods for more details.

Among those 51 measures, 6 were significantly predictable (see Materials and Methods) and achieved a mean Pearson's correlation of >0.15 between the predicted and true behavioral scores across all test participants, including every ethnic/racial group. The correlation accuracies on the entire test sets can be found in fig. S3A. Among these six behavioral measures, five showed significantly larger predictive COD in WA than AA: spatial orientation, grip strength, reading (pronunciation), cognitive flexibility (Dimensional Change Card Sort), and openness [Neuroticism/Extroversion/Openness Five Factor Inventory (NEO)] (Fig. 3A). The false discovery rate (FDR) was controlled at 5% across the six behavioral measures to correct for multiple comparisons. Considering all 51 behavioral measures, i.e., also those that did not show a significant prediction in both groups, 42 of them showed significantly larger predictive COD in matched WA than in AA (fig. S4A). No behavioral measure exhibited significantly lower predictive COD in WA than AA.

Next, we looked into the direction of prediction errors of AA and WA for each behavioral measure separately (fig. S6A). A wider range of the difference between predicted and original behavioral scores in WA than AA was observed due to the fact that different sets of WA were selected to match the same set of AA across 40 random repetitions of splits. This complicated the examination of differences between the two populations in HCP. Considering this limitation, overall, some behavioral scores were more overpredicted (i.e., predicted higher than actual) in AA than WA—particularly visual episodic memory, sustained attention, cognitive status [Mini Mental State Examination (MMSE)], arithmetic, four of the NEO Big Five measures (except conscientiousness), fear, sadness, positive affect, and two social support measures—while other scores were more underpredicted (i.e., predicted lower than actual) in AA than WA, namely, grip strength, taste intensity, anger—aggression, conscientiousness (NEO), and perceived rejection.

ABCD dataset

For the ABCD dataset, because of the large discrepancy of sample size across sites, we used the following data matching and split procedures (Fig. 2B). First, within each site, Hungarian matching was performed to select AA and WA pairs that had minimal differences in confounding variables and behavioral scores. Second, sites were combined into 10 sets, based on a balanced number of matched AA-WA pairs across the 10 sets, to achieve similar test sample sizes during cross-validation. Last, for each cross-validation iteration, 7 of the 10 sets were chosen as the training set and the remaining 3 folds were chosen as the test set. The behavioral distributions of matched AA and WA can be found in fig. S2.

Nine of the 36 behavioral variables showed predictability and achieved >0.15 correlation accuracy across the whole test set (Fig. 3B and fig. S3B). Four behavioral measures exhibited significantly higher predictive COD in WA than AA: crystallized cognition, working memory (list sort), picture vocabulary, and executive function (card sort) (FDR controlled at 5% across the nine behavioral measures). On the contrary, only matrix reasoning showed significantly lower predictive COD in WA than AA. When all 36 behavioral measures were considered, 25 of them showed significant predictive COD difference between the matched WA and AA (fig. S4B). Twenty of the 25 behavioral measures exhibited higher predictive COD in WA. In comparison, the other five measures [visual episodic memory, anxious/depressed feelings, matrix reasoning, somatic complaints, and Behavioral activation system (BAS)—reward responsiveness] exhibited higher predictive COD in AA.

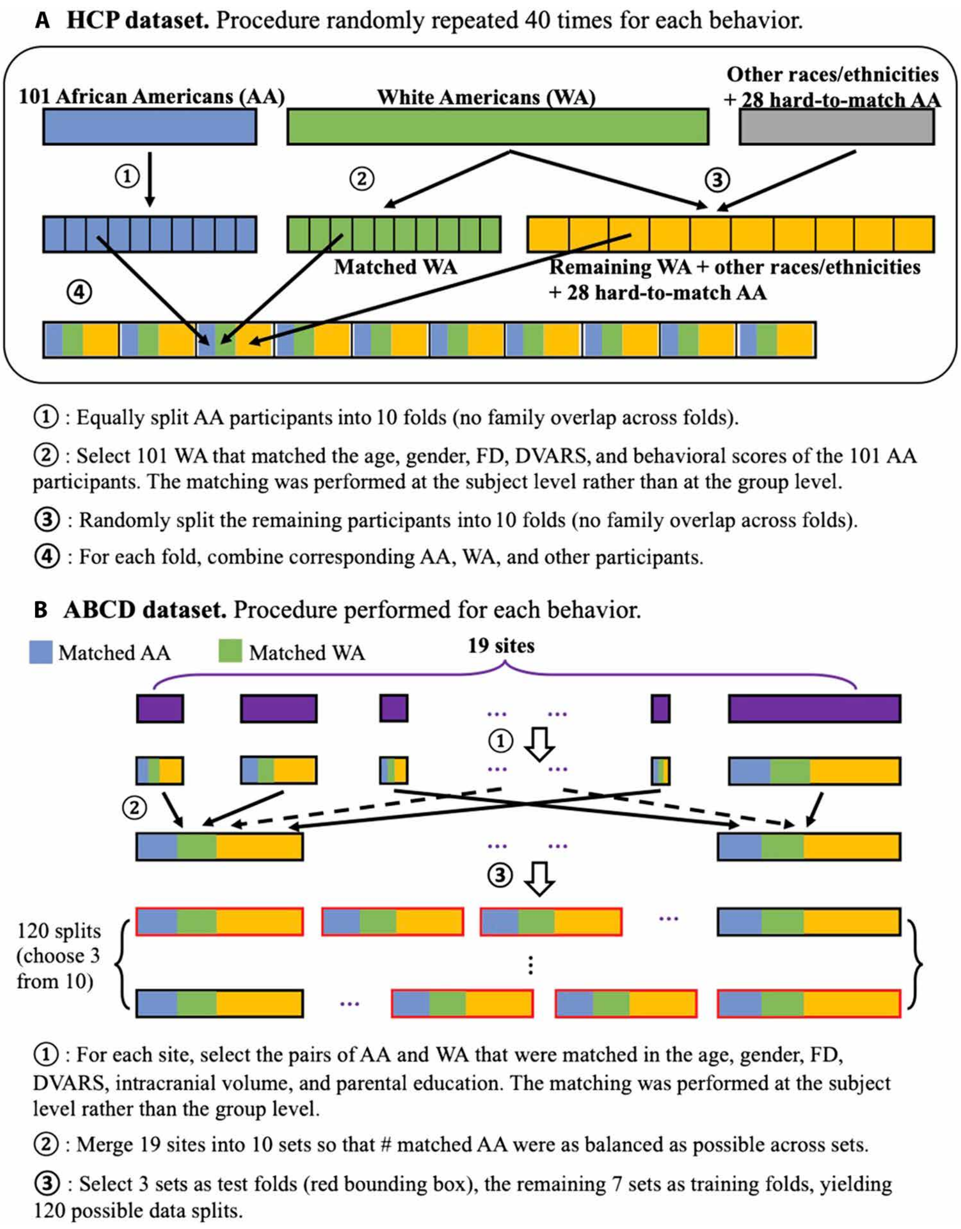

Fig. 2. Procedures for data splitting and the matching between WA and AA. (**A**) HCP dataset. (**B**) ABCD dataset.

Overall, cognitive scores were more underpredicted in AA compared to WA in this dataset (fig. S6B), e.g., short-/long-delay recall, cognitive control/attention (flanker), working memory (list sort), executive function (card sort), crystallized cognition, and overall cognition. In contrast, psychometric scores such as somatic complaints, social problems, rule-breaking behavior, aggressive behavior, positive urgency, and behavioral inhibition tended to be more overpredicted in AA than WA.

Full-dataset model (linear ridge regression) yielded higher prediction error in AA than in WA

To ensure that the larger prediction error in AA than WA was not algorithm specific, we used linear ridge regression as a complementary method to corroborate the main findings. In the HCP dataset, seven behavioral measures were predictable and achieved >0.15 correlation accuracy across the whole test set (Fig. 3C). All seven measures showed significantly higher predictive COD in WA than AA (e.g., anger—aggression and spatial orientation). Across all 51 behavioral measures, 44 measures exhibited significantly larger predictive COD in WA than AA (fig. S6A). For the remaining seven measures {e.g., sleep quality [Pittsburgh Sleep Quality Index (PSQI)] and inhibition (flanker task)}, no significant difference in test predictive COD was found between AA and WA.

In the ABCD dataset, seven behavioral measures were predictable and achieved >0.15 correlation accuracy across the whole test set (Fig. 3D). Four of these seven measures showed significantly larger predictive COD in WA than AA: picture vocabulary, working memory (list sort), matrix reasoning, and crystallized cognition. The remaining three measures did not show any significant difference. Among all 36

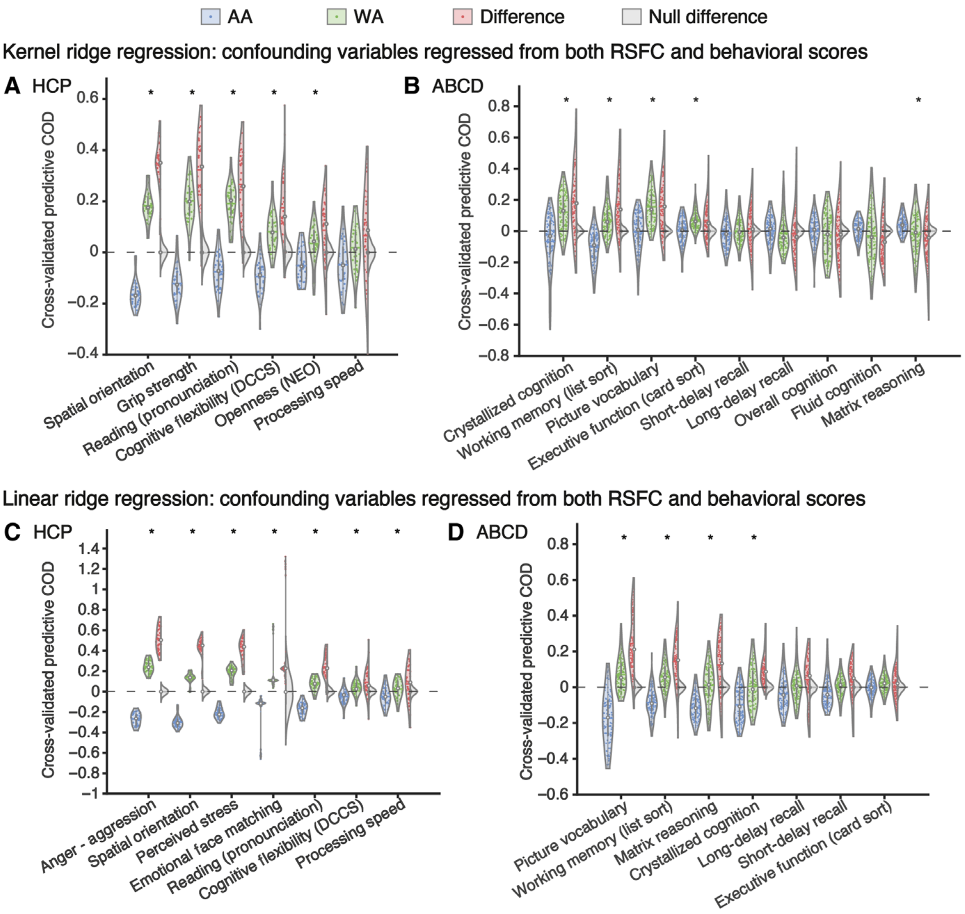

Fig. 3. The prediction error is larger (i.e., lower predictive COD) in AA than matched WA in the HCP dataset (left) and the ABCD dataset (right). Each violin plot shows the various predictive COD across 40 data splits in (**A**) and (**C**) and across 120 training-test splits in (**B**) and (**D**). Blue and green violins represent AA and WA, respectively. Red violins are the difference. Gray violins show the null distribution of difference generated by flipping the AA versus WA labels. Asterisk indicates that the difference in predictive COD between matched AA and WA is significant (FDR controlled at $q = 5\%$). Gray dashed line indicates 0.

behavioral measures, 23 measures exhibited significantly larger predictive COD in WA than AA, e.g., visuospatial reaction time (fig. S6B). Only total prodromal psychosis symptoms showed significantly lower predictive COD in WA than AA.

In general, the overall prediction accuracy and the difference between AA and WA were replicated using linear ridge regression (compared to using kernel ridge regression). For the following analyses, we focused on kernel ridge regression.

Effects of confound regression on prediction error bias

As different distribution of confounding variables across ethnic/racial groups might influence the observed differences between WA and AA in prediction results in an unexpected way, we further compared the prediction accuracy between AA and WA without confound regression. In the HCP dataset, 15 behavioral measures showed predictability and achieved >0.15 correlation accuracy across all test participants (Fig. 4A). For 12 of these 15 measures, predictive COD was significantly higher in WA than AA, e.g., anger—aggression and reading (pronunciation). For the remaining three measures [working memory (list sorting), sleep quality (PSQI), and inhibition (flanker task)], difference in predictive COD between AA and WA was not significant. Among a total of 51 behavioral measures, 45 measures exhibited significantly larger predictive COD in WA than AA, e.g., contrast sensitivity and social cognition—interaction (fig. S7).

In the ABCD dataset, there were also 15 behavioral measures that were predictable and with >0.15 correlation accuracy across the whole test set (Fig. 4B). Six behavioral measures showed significantly higher predictive COD in WA than AA, e.g., cognitive control/attention (flanker) and working memory (list sort). In contrast, three behavioral measures exhibited significantly lower predictive COD in WA than AA: visual episodic memory, matrix reasoning, and overall cognition. Among all 36 behavioral measures, 20 of them showed significantly larger predictive COD in WA than AA, e.g., visuospatial reaction time and social problems (fig. S7). On the contrary, seven measures showed higher predictive COD in AA than WA, e.g., visual episodic memory and anxious/depressed.

In summary, without confound regression, as expected, prediction accuracy was generally higher than that with confound regression. Hence, more behavioral measures were included in Fig. 4 compared to Fig. 3, regardless of whether the predictive COD was different between AA and WA. Overall, however, the set of behavioral measures showing significant differences in prediction error (independently of direction) largely overlapped, and our general finding of better-performing models in WA remained.

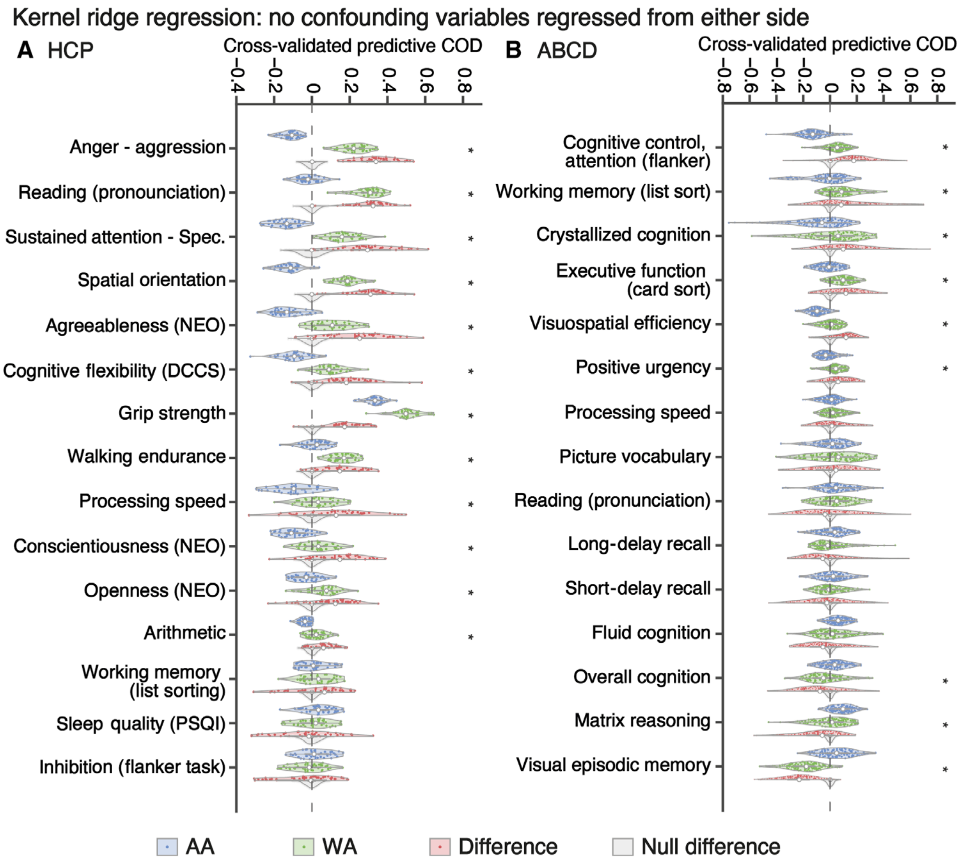

Fig. 4. Predictive COD of AA and WA when no confounding variable was regressed during model building. (**A**) HCP dataset. Each violin plot shows the various predictive COD across 40 data splits. (**B**) ABCD dataset. Each violin plot shows the various predictive COD across 120 training-test splits. Blue and green violins represent AA and WA, respectively. Red violins show the difference. Gray violins show the null distribution of difference generated by flipping the AA versus WA labels. Asterisk indicates that the difference in predictive COD between matched AA and WA is significant (FDR controlled at 5%). Gray dashed line indicates 0.

Training the model specifically on AA increases its performance for this population

Since the full datasets were dominated by WA participants, an intuitive solution to obtain a more valid predictive model for a specific minority group, for which insufficient generalizability was observed, would be to perform the training on this minority. Using the ABCD dataset, we trained kernel ridge regression on all AA participants. As a comparison, kernel ridge regression was also trained on the same number of randomly selected WA participants, as well as on an equal number of AA and (randomly selected) WA participants. These three types of models were then tested on matched AA and WA separately as above. This analysis could not be conducted for the HCP dataset because the number of AA in HCP was insufficient for building reliable predictive models.

For the model trained solely on AA, 19 of the 36 behavioral measures showed significantly higher predictive COD when this model was tested on matched WA than when tested on matched AA (Fig. 5A). Conversely, the AA-trained model achieved higher predictive COD in AA than WA for eight behavioral measures. No significant difference was observed for nine behavioral measures. In contrast, for the model trained solely on the same number of random WA, higher predictive COD in WA than AA was observed for 26 behavioral measures. Last, the WA-trained model only performed better, in terms of predictive COD, in AA than WA for two behavioral measures. Differences in predictive COD between AA and WA were not significant for eight behavioral measures using the WA-trained model. A detailed list of behavioral measures for which significant and nonsignificant differences were observed is reported for the different approaches in Table 1.

The model trained on equal numbers of AA and WA resulted in AA-WA accuracy differences that were in between the former two modeling approaches (AA only and WA only). Specifically, 24 behavioral variables exhibited higher predictive COD in WA than AA, and 5 behavioral measures showed lower predictive COD in WA than AA. Overall, this suggested that strategies on training population could ameliorate but not eliminate the accuracy difference between the two groups. More concretely, training on AA improved the out-of-sample accuracy of AA, compared to training on a WA sample or a balanced mixed population [e.g., for picture vocabulary, working memory (list sort), overall cognition, fluid cognition, short-delay recall, long-delay recall, BAS—reward responsiveness, and visual episodic memory].

Different brain-behavior associations learned from AA only versus from WA only

Given the different performance of prediction models trained on different subpopulations, we investigated more deeply on whether these models learned different associations between RSFC and behavioral data.

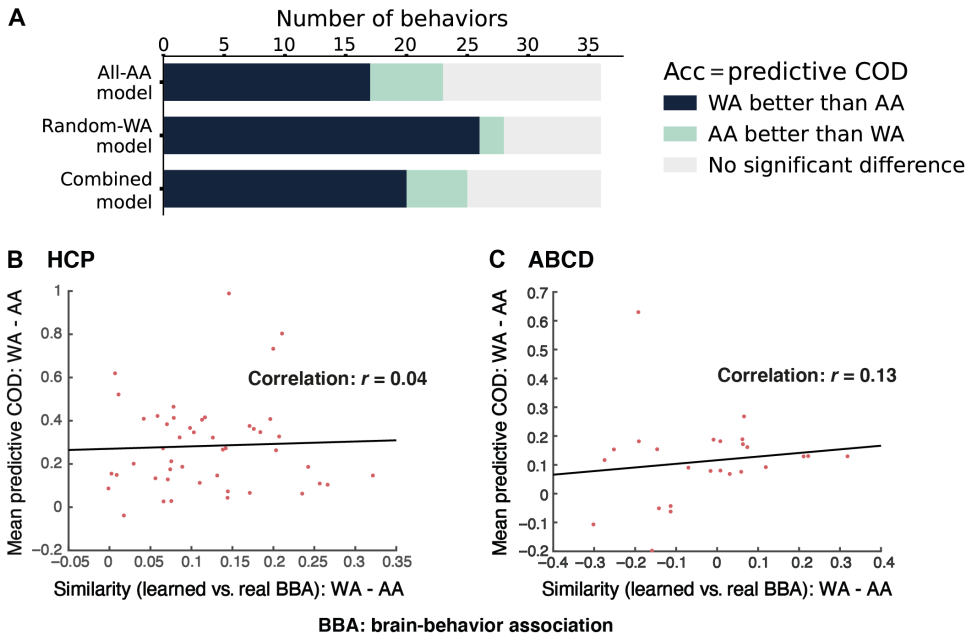

Fig. 5. Extended analyses. (**A**) Impact of training population for behavioral prediction model. The influence of training population was evaluated using predictive COD. Each bar corresponds to one of three types of prediction models: (i) trained on AA only, (ii) trained on the same number of random WA, and (iii) trained on both. For each model, the number of behavioral measures with better performance in WA than AA is indicated by the length of the navy blue bar, while the mint color represents the number of behavioral variables with better performance in AA than WA. The gray color represents the number of behavioral variables not showing significant difference in test accuracies between AA and WA. (**B** and **C**) For full-dataset models (when models were trained on the entire dataset), plot AA versus WA accuracy difference (predictive COD; vertical axis) against the difference in similarity between model-learned brain-behavior association patterns and true groupwise brain-behavior association patterns (horizontal axis). Each red dot represents a behavioral measure.

We quantified the brain-behavior association learned by the models as the covariance between the predicted behavioral scores and the RSFC between each pair of brain regions across the training participants (*34*). Overall, although similar patterns of model-learned brain-behavior associations could be observed by training only on AA versus training only on WA, broad patterns of discrepancies could easily be seen (figs. S8 and S9). For example, models trained on AA only learned negative associations between visual A–limbic B RSFC and behavioral measures including short-delay recall, long-delay recall, processing speed, visual episodic memory, fluid cognition, and overall cognition. However, this pattern did not appear when models were trained on WA only. For short-delay recall and long-delay recall, the WA-trained models even learned positive relationship between this RSFC feature and behavioral scores. Another example of discrepancy can be observed in fig. S9. For most behavioral measures in this figure (e.g., processing speed), a relatively strong positive association between the RSFC of the somatomotor network and behavioral scores was observed for the models trained only on WA, while this pattern was much weaker for the models trained on AA. For measures such as visual episodic memory and visuospatial accuracy, models trained on WA learned much simpler, focused patterns of brain-behavior negative association, compared to the models trained on AA. Although further investigations are needed to explain these different patterns of brain-behavior association learned from AA and WA, the current findings provided some insights on the different prediction accuracy biases of models trained on different ethnic/racial groups.

More similar model-learned versus true brain-behavior associations in the higher-accuracy group

Coming back to the original models trained on the sample mixing all ethnic/racial groups, an important question raised by the differences in model accuracy pertains to the validity of the learned brain-behavior associations. Concretely, we investigated whether the prediction model learned more valid representations of brain-behavior association (i.e., the learned associations being better corresponding to the true one) for the subpopulation with higher accuracy than the other one. Similar to the previous section, model-learned brain-behavior association was calculated, but, here, for the full-dataset models, while the true brain-behavior association was defined as the covariance between the true behavioral scores and each element of the RSFC matrix across test participants. The similarity between the model-learned and true brain-behavior association across all region-to-region pairs in AA and WA separately was then computed as Pearson's correlation.

Overall, behavioral measures showing more similar model-learned versus actual brain-behavior association patterns in one ethnic/racial group than the other tended to have greater accuracy in the former group as illustrated in Fig. 5 (B and C). In this figure, higher value on the horizontal axis means that model-learned brain-behavior association was more similar to the true brain-behavior association in WA than AA [i.e., similarity(model-learned association patterns versus true patterns in WA) − similarity(model-learned association patterns versus true patterns in AA)]. The vertical axis represents how much the predictive COD of WA exceeded that of AA. A positive, although weak, relationship between the two measures

across all behavioral variables was observed in both datasets. This suggests that the model tended to learn brain-behavior association patterns that were more valid for the subpopulation with the higher prediction accuracy (in most cases, it was WA).

Consistency/inconsistency across prediction accuracy metrics

In previous sections, we focused on the accuracy as assessed with the predictive COD. In this study, the lower predictive COD of a specific group (e.g., AA) indicated the larger deviation of predicted behavioral scores from the true behavioral scores of the same group (i.e., MSE), by a factor of the respective true behavioral variance in the training set (see Materials and Methods). Since Pearson's correlation has been and is still frequently used for evaluating the performance of brain-behavior predictive models (*18*, *35*–*37*), we also repeated our analyses with Pearson's correlation as a prediction accuracy metric. While predictive COD quantifies the individual differences between the predicted and observed values, Pearson's correlation focused on the overall linear relationship between predicted and observed behavioral scores (i.e., co-increase/decrease of predicted and true behavioral scores).

First, we compared the out-of-sample correlation accuracy between AA and WA, when predictive models were trained on the entire datasets (fig. S10). Overall, most of the predictable behavioral measures (yellow text color in the figures) exhibited higher correlation accuracy in WA than in AA, although some behavioral measures showed the opposite pattern, including grip strength in the HCP dataset and visual episodic memory, long-delay recall, and matrix reasoning in the ABCD dataset. When considering the whole range of 51 behavioral measures in the HCP data and the 36 behavioral measures in the ABCD data, a pattern of higher predictive COD in WA than in AA but with lower correlation accuracy in the former than the latter was observed for several variables (fig. S4 versus S10).

To further explore the possible reasons for the discrepancies between the two metrics, we examined the scatterplots of predicted scores against true scores for the behavioral variables showing inconsistency between the two metrics (Fig. 6). First, one should note that the calculation of the predictive COD is based on a common behavioral variance shared across AA and WA, assuming that the behavioral variance should not be group-specific (see Materials and Methods). Hence, predictive COD and MSE formed an inverse relationship, i.e., the larger the MSE, the smaller the predictive COD. In reality, we observed larger behavioral variance in AA than in WA for some behavioral measure (Fig. 6, A, C, and E). In the HCP dataset, different variances of true behavioral scores between the matched AA and WA were observed by conducting Levene's test (FDR controlled at 5%) for cognitive status (MMSE), social cognition—random, social cognition—interaction, emotion recognition—happy, anger—affect, anger—hostility, anger—aggression, fear—affect, positive affect, loneliness, perceived rejection, emotional support, and instrument support. In the ABCD dataset, although the variances differed numerically, these differences were not significant using Levene's test (FDR controlled at 5%). The larger behavioral variance in AA could lead to higher MSE for AA than WA, i.e., lower predictive COD for AA than WA. Second, we observed a larger overall prediction shift (i.e., the deviation of average predicted behavioral score from average true behavioral score) in AA than in WA for some behavioral measures (Fig. 6, B, D, and F). In other words, a systematic bias appears in the prediction of AA, with the predicted behavioral scores being

Table 1. List of behavioral measures with better prediction in WA, with better prediction in AA, or without significant difference in prediction between WA and AA, for the models trained only on AA and only on WA.

	Predictive COD: WA > AA	Predictive COD: WA < AA	No significant difference
Model trained only on AA	•Visuospatial reaction time	•Short-delay recall	•Visual episodic memory
	•Reading (pronunciation)	•Total prodromal psychosis symptoms	•BAS—fun seeking
	•Visuospatial efficiency	•Anxious/depressed	•Executive function (card sort)
	•Thought problems	•Rule-breaking behavior	•BAS—drive
	•Social problems	•Somatic complaints	•Withdraw/depressed
	•Behavioral inhibition	•Long-delay recall	•Visuospatial accuracy
	•Mania	•Attention problems	•Prodromal psychosis severity
	•Matrix reasoning	•BAS—reward responsiveness	•Processing speed
	•Aggressive behavior		•Picture vocabulary
	•Crystallized cognition		
	•Positive urgency		
	•Fluid cognition		
	•Cognitive control/attention (flanker)		
	•Lack of perseverance		
	•Lack of planning		
	•Sensation seeking		
	•Working memory (list sort)		
	•Overall cognition		
	•Negative urgency		

continued on next page

	Predictive COD: WA > AA	Predictive COD: WA < AA	No significant difference
Model trained only on WA	•Picture vocabulary<br>•Working memory (list sort)<br>•Visuospatial reaction time<br>•Reading (pronunciation)<br>•Crystallized cognition<br>•BAS—drive<br>•Aggressive behavior<br>•Overall cognition<br>•Visuospatial efficiency<br>•Thought problems<br>•Sensation seeking<br>•Withdrawn/depressed<br>•Behavioral inhibition<br>•Matrix reasoning<br>•Cognitive control/attention (flanker)<br>•Lack of perseverance<br>•Social problems<br>•Long-delay recall<br>•Short-delay recall<br>•Negative urgency<br>•BAS—reward responsiveness<br>•Visuospatial accuracy<br>•Lack of planning<br>•Positive urgency<br>•Visual episodic memory<br>•BAS—fun seeking	•Total prodromal psychosis symptoms<br>•Anxious/depressed	•Somatic complaints<br>•Attention problems<br>•Executive function (card sort)<br>•Fluid cognition<br>•Mania<br>•Rule-breaking behavior<br>•Processing speed<br>•Prodromal psychosis severity

consistently lower or higher than the true behavioral scores, to a larger extent than in WA. The larger prediction shift of AA would also lead to higher MSE and thus lower predictive COD. However, such prediction shifts/systematic prediction bias in AA are not captured by the correlation between the predicted and true scores, yielding higher correlation accuracy in AA than in WA. In the HCP dataset, 13 behavioral measures showed inconsistent conclusions across the two accuracy metrics. Six of them exhibited a larger prediction shift in AA than in WA. Within these six behavioral measures, grip strength and anger—aggression showed a more negative prediction shift in AA than in WA, while for the remaining four measures (sustained attention—Specificity, social cognition—random, positive affect, and emotional support), the predicted behavioral scores of AA were more positively shifted than those of WA. In the ABCD dataset, attention problems, prodromal psychosis severity, mania, thought problems, and BAS—drive showed inconsistent results across the two accuracy metrics. Prodromal psychosis severity, mania, and BAS—drive exhibited a larger prediction shift in AA than in WA. For all these three measures, AA's scores were more overpredicted compared to WA. In summary, larger behavioral variance and a pattern of prediction shift/systematic prediction bias in AA can lead to higher prediction errors in AA captured by predictive COD but not by the correlation metric.

Beyond these observations, follow-up analyses using Pearson's correlation corroborated the observations reported in the previous section. Overall, our findings on how altering training population influences the model performance difference between AA and WA were replicated (fig. S11A). Furthermore, better prediction performance in one ethnic/racial group than the other, as measured by Pearson's correlation, was also related to the higher validity of the model-learned brain-behavior association patterns for the former group in the ABCD dataset (fig. S11, B and C).

DISCUSSION

The prediction of behavioral phenotypes from brain imaging data, especially from fMRI data, is a topic currently undergoing intense study as it holds high promise for applications in cognitive and clinical neuroscience (*18*, *38*, *39*). With potential successful predictive models in the future, the association of brain functional organization with behavioral phenotypes could be understood at the individual level rather than at the group level. It will also contribute to the development of markers for preventing, diagnosing, and treating mental disorders. However, successful predictive methods should not only achieve overall high accuracy but also have an equal level of validity for different (sub-) populations. Given the fact that most

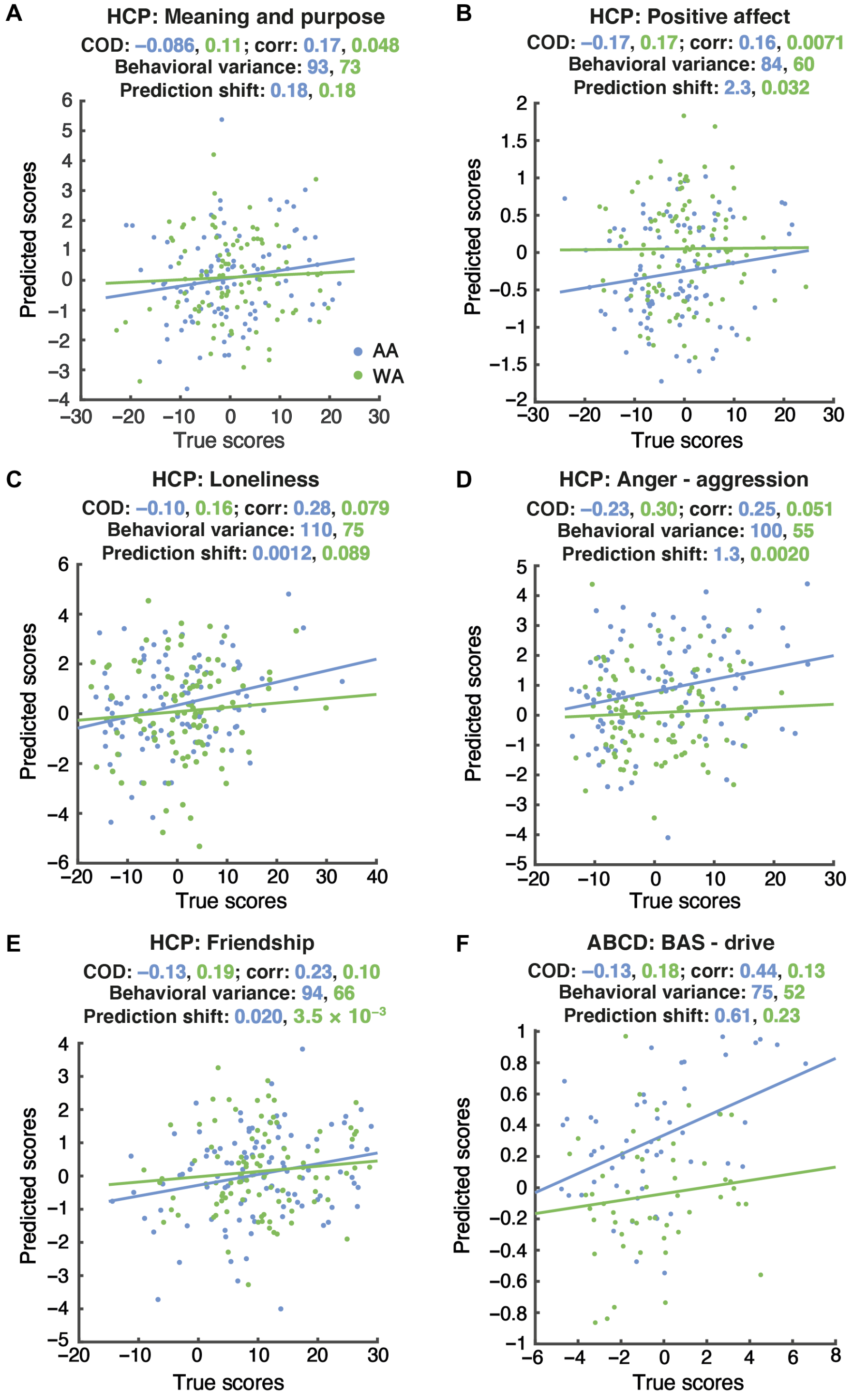

Fig. 6. Scatterplots of predicted scores against true behavioral scores for the behavioral measures with inconsistent conclusion drawn from predictive COD and Pearson's correlation. For each behavioral measure, a representative data split is shown (**A** to **F**). Each blue or green dot represents one AA or WA test participant, respectively. The numbers reported in blue color correspond to AA, while the green ones correspond to WA. Behavioral variance refers to the variance of the true behavioral score. Prediction shift was calculated as the square of the mean difference between true and predicted scores.

of the recent research on behavioral prediction focused on datasets largely formed by the white population, we raised the question whether the models trained on such samples could perform equally well on other ethnicities/races compared to white populations. To answer this question, we examined the difference in prediction performance between white/European Americans and AA of models trained on a mixture of multiple ethnicities/races, following the dominant approach currently taken in the field. Our study was performed on two high-quality large-scale datasets, which had different age ranges, scanning protocols, and preprocessing strategies.

Therefore, the issues pertaining to model generalizability appear replicable and independent of the dataset.

In summary, we observed significantly lower predictive COD (hence larger prediction error) in AA than in WA for most behavioral measures, using two types of machine learning algorithms, when the models were trained on the entire datasets, i.e., a training set including all ethnicities/races but dominated by individuals of European ancestry. When the models were trained solely on AA, the test AA participants enjoyed better accuracies, compared to the models trained on the same number of WA or on a mixed but balanced samples of AA and WA. Nevertheless, training the model specifically on AA did not eliminate the accuracy differences between AA and WA.

Technical choices and their related potential limitations

All aforementioned results were tested on matched AA and WA for a diverse set of variables. Matching is necessary to control for a different data structure in a different population on the prediction accuracy (for example, different range for the target variable in different populations). However, given the covariation of ethnicities/races and demographic, morphologic, or psychometric measures, some participants were omitted during matching. For example, because of societal reasons, AA tended to receive fewer years of education than WA. After matching, poorly educated AA and extremely educated WA were not considered for the latter comparison. While necessary for the current investigation, we note that this approach may limit the generalizability of any model in the case of strong and true stratification by confounds (*40*). That is, if the distribution of a confound is largely nonoverlapping, focusing on only the low and high end, respectively, will not be representative of the underlying population. Challenges raised by such systematic differences across populations need to be further addressed in the future. Furthermore, different matching approaches could be considered [such as propensity score matching (*41*) and coarsened exact matching (*42*)], and their effects on the downstream prediction differences should also be investigated in the future.

Similarly, in our main analyses, several variables were selected as possible "confounders" and were regressed out from both brain and behavioral data. Note that we use the more liberal definition of confounders taken from statistics, which here refers to the variables that are generally correlated with both brain and behavioral data. In other words, we do not assume specific causal direction from a conceptual standpoint when referring to them as confounders. We would argue that the causal relationships among these variables, brain and behavioral measures, are still under active investigation. Nevertheless, to ensure that our findings were not affected in an unexpected way by a relatively blind confounds removal, we repeated our main analyses without regressing such variables. We could hence show that our results could be replicated when refraining from confounds removal.

Beyond AA, the generalizability of predictive models in other minority populations should also be investigated. In particular, the Hispanic population also represents a minority with long-term unprivileged socioeconomic status in U.S. datasets (*43*, *44*). The relatively large number of Hispanic participants in U.S. datasets, such as the ABCD dataset, may, however, allow a rigorous investigation of this question in future studies. Other minority groups should also benefit from generalizability investigations, such as native Americans. However, the currently low number of participants in open datasets may complicate the implementation of a proper cross-validation scheme in these populations.

Although two independent datasets were used in this study, predictive models were trained and tested within each dataset separately. Cross-ethnicity/race biases were not investigated for cross-dataset prediction considering the length of this article. However, it should be acknowledged that the generalizability of behavioral prediction models across datasets is a crucial research topic and is still under intensive investigation (*20*, *45*). How predictive models trained in one dataset could generalize to multiple ethnic/racial groups in another dataset should be examined in the future.

Acknowledging these potential limitations and the need for further investigations in the future, in the following paragraphs, we discuss the implications of our current findings for data collection and data analysis strategies. We also briefly warn against neuroscientific misinterpretation and address the possible downstream consequences of our findings.

Calling for data collection from non–European-descended populations

The two datasets used in this study are among the largest datasets of neuroimaging and extensive behavioral phenotyping in the field and hence are among the most valuable resources for brain-behavior predictive model. However, both datasets are predominately formed by white/European individuals: 76% in the HCP dataset and 56% in the ABCD dataset (Fig. 1). As an additional promising resource for neuroimaging-based predictive models, the currently largest dataset with neuroimaging measurements is the UK Biobank dataset (*24*). However, this sample is also mainly formed by European descendants (97.3%). While the second-largest ethnic/racial group is Asian British, it represents only 1% of the cohort, resulting in around 300 participants for which both fMRI and behavioral data are available. These datasets were thoughtfully collected to represent the ethnic distribution in the local population. Yet, the current sample sizes of minorities are not sufficient to achieve adequate levels of performance for training predictive models from minority groups. In the current study, the sample-size limitation of AA also prevents us from matching them with WA more accurately so that the difference in prediction accuracies could be more comprehensively evaluated. From this perspective, our findings urge for more neuroimaging and behavioral data collected from non-European/white ethnic/racial groups.

Moreover, we need to be aware that the U.S. perspective is dominating our recognition and categorization of ethnicities and races, at least in the field of neuroscience. For instance, although Chinese and Indian populations are largely different regarding cultures, diets, etc., they were often treated together as "Asian." Similarly, people from the Middle East cannot be easily classified as either white/European or Asian. In addition, although current datasets such as the HCP dataset were dominated by white/European participants, they cannot be assimilated to the population in the European continent, not to mention the diversity within the European population. It is important to design studies that include a complete global spectrum of ethnicities, beyond the U.S. ethnic categorization, to fully understand human brain and behavior. Looking into each specific non-U.S. population, care should also be taken to avoid sampling problems as in the current U.S. datasets. Taking one of the rising scientific communities, the Chinese population, as an example, minority ethnicities within China except the Han Chinese are often

neglected or underrepresented in data collection, leading to a lack of investigation in non-Han Chinese ethnicities. Thus, the issue of underrepresentation of minorities in neuroscience studies and applications goes importantly beyond the specific population investigated in this study.

A fairness perspective with limited neuroscientific insight

The present study has been designed from the perspective of assessing and promoting fairness of future artificial intelligence applications across different subgroups of a population. This question was addressed from a societal standpoint targeting specific social groups, hence based on self-reported information rather than evolutionally defined ancestral information. Moreover, as explained above, valid assessment required the two groups to be matched regarding behavioral performance. Consequently, the results reported in this study do not allow any insight into possible neurobiological or neurocognitive differences between the investigated groups. In particular, nature and nurture can hardly be disentangled with the present data, and the interpretation of our results from a basic neuroscientific standpoint is clearly limited. For instance, disparities in public sector investments in education (*46*, *47*), family structure and socioeconomic status, and peer influence could mediate the educational resources of different ethnic/racial groups (*48*). Complex models of ethnic/racial differences in educational achievement considering both structural and cultural inequality have hence been proposed (*49*). Furthermore, from a neuroscience perspective, socioeconomic status was also shown to be related to brain morphometry in adolescents and to moderate brain activity during cognitive and emotion processing (*27*, *50*). Last, from a medical perspective, ethnic/racial minorities tended to have less access to health care and receive lower quality of health care, which, in turn, lead to poorer health outcomes, i.e., health disparities. Thus, many aspects of structural disparities should be considered for phenotype prediction with the goal of potentially influencing related policy making. We thus believe that neuroscientific studies should focus efforts on identifying efficient strategies for reducing biases in future technologies rather than developing neurobiological theories about group differences that potentially contribute to neuroracism (*51*). New study designs, equipment, and preprocessing strategies should be proposed to highlight methodological bias in past literature of group differences, adapt to the diversity of the population, and build fairer artificial intelligence for the future.

Downstream consequences of prediction disparity across ethnicities/races

In addition to our examination of overall prediction accuracies between AA and WA, we also looked into the direction of prediction error in AA and WA for each behavioral measure. In the HCP dataset, we observed more positively predicted social support measures (emotional support and instrumental support) and more negatively predicted social distress measures (perceived hostility and perceived rejection) in AA than WA (fig. S5A). Therefore, one could be concerned that by uncritically relying on the model prediction, the pressure that AA feel may be underestimated while the support they receive during social interactions may be overestimated. Some patterns found in the ABCD dataset further raised potential societal and ethical consequences if the predicted target variables were used without further inspection. For example, for most measures in the Achenbach Child Behavior Checklist (social problems, rule-breaking behavior, etc.), higher predicted scores compared to observed scores were found, to a larger extent, in AA than WA. This means that more social problems and rule-breaking behavior were predicted in AA than WA, compared to the actual behavior (fig. S5B). However, behavioral aspects such as aggressive and rule-breaking behaviors can contribute to a mental disorder diagnosis. An overestimation of behavioral troubles based on the pattern of brain connectivity could hence yield to more false positives if the diagnosis of disorders was merely made by machine learning algorithms. Thus, our finding points to the need of refinement and resolution of specific, potentially detrimental, predictive biases before similar model frameworks are deployed in artificial intelligence–based diagnosis systems.

Limitation of studying ethnicity/race inequality in a counterfactual framework

In the context of counterfactual causal inference, the findings of the current study should be interpreted as a behavioral prediction disparity between AA and WA that would remain when the distributions of basic demographic, anthropometric, and scan-related measures were equalized between the two groups (*52*), given that these measures were matched between test AA and WA. As we mentioned above, matching was performed to avoid effects that would be attributable to differences in these confounding variables (e.g., prediction accuracy might be different between males and females even within the same ethnicity/race). Although we matched the test samples for comparison in our work, matching on a few available potentially confounding variables does not appear to be an optimal strategy in this applied research setting.

In the context of ethnical/racial inequality, limitations of the counterfactual framework have been raised recently (*53*). The major criticism was that ethnicity/race could not be manipulated. Since ethnicity/race is a complex concept that integrates a myriad of aspects such as physical appearance, culture, and socioeconomic status, it is not possible to manipulate the race status of otherwise identical units (e.g., gestational environment, neighborhood, self-perception, and the effects of other perception in social interactions). In other words, it is not reasonable to imagine an experimental setting in which an AA and a WA are made similar in all aspects except for race at the testing time t. Because appropriate solutions at the algorithmic/technical level are still under investigation, here, we followed a traditional statistical approach but acknowledge this as an important limitation of our study.

Multiple model performance metrics are necessary

When the predictive models were trained on the sample with all ethnicities/races, the WA exhibited a significantly higher out-of-sample predictive COD than the matched AA for most behavioral phenotypes, suggesting lower prediction error in WA than AA (Fig. 3 and fig. S4). The accuracy measured by Pearson's correlation also showed significant differences between the two subpopulations, although for some behavioral measures, the correlation of AA was greater than that of WA (fig. S10). We therefore looked into the possible factors contributing to such discrepancies across accuracy metrics. For the behavioral measures showing discrepancies between the two metrics, we observed a larger prediction shift and/or a higher variance of the original behavioral score in AA as compared to WA participants. A larger prediction shift refers here to a systematic prediction bias, which means that the model predicted the scores of

AA with a systematically larger error (either positive or negative) from the original scores. It is possible that the models learned behavioral scores that were more similar to WA on average, since the training sets were dominated by WA. The model-learned average behavioral scores might be different from the average true behavioral scores of AA. However, as a model performance metric, Pearson's correlation ignores that shift or systematic bias. Thus, on the one hand, Pearson's correlation has some limitations as an indicator of model performance. However, on the other hand, accuracy metric based on individual differences between the predicted and true scores may also show limitations in a specific situation. In this study, although we matched AA and WA as much as possible, the behavioral variances of AA were still generally larger than that of WA, especially for the HCP dataset due to the relatively limited AA sample size. Probably due to fewer data points at distribution tails compared to the center, predictive models tend to overestimate the lower values and underestimate the higher values. Therefore, larger variance in the original behavioral scores (i.e., more data points on the tails) was related to larger prediction error and lower predictive COD. Overall, these results indicate that the two common metrics capture different aspects of the model performance, are sensitive to different structural aspects of the original data, and, hence, should be used in combination for a greater insight into the model evaluation as recently suggested in the field (*33*).

Other possible factors contributing to the differences in prediction performance

To investigate the impact of training population, we examined the performance of predictive models trained specifically on either only AA or only WA. By doing so, we found that, although prediction in AA benefited, to some extent, from specific AA training compared to specific training on only WA, some significant AA-WA differences in the test accuracies were still observed (Fig. 5A and fig. S11A). Beyond the composition of training samples, potential factors pertaining to data acquisition and preprocessing might contribute to cross-population generalization failures.

Considering brain data, an important preprocessing step is to align individual scan to a common space/template so that different participants can be compared or averaged. However, the commonly used volumetric and surface templates such as MNI152 (a standard template generated by Montreal Neurological Institute), fsaverage, and fs_LR (also known as Conte69) were mostly built on white/European-descendant participants. This raises the question of the validity of the preprocessing of the data of non-European populations based on these templates. Recent studies have emphasized the need for a population-specific template in neuroimaging studies, such as Chinese-specific templates. Several morphological differences between Caucasian templates and the templates built on large-scale Chinese populations have hence been suggested, given that the use of Chinese-specific templates has led to higher segmentation accuracy and smaller shape deformations in the Chinese sample than Caucasian templates (*54*, *55*). Nevertheless, to the best of our knowledge, specific African templates have not been built and evaluated for the preprocessing of African population brain data. Thus, future work could focus on creating appropriate African templates and investigate the effect of template selection on behavioral prediction. Another possible source of bias could be functional atlas, as the cortical parcellation we used here was derived from a dataset that was also predominately composed of WA (*56*). In that regard, it has been shown that functional parcel boundaries could vary across individuals and even across brain states (*57*, *58*). Therefore, a relevant question for future research is whether adopting individualized functional brain parcellations, or, at least, a parcellation that is representative of an African population atlas, would reduce performance inequality in subsequent predictive modeling. In this study, we focused on RSFC as the predicting neural phenotypes. However, generalization failure of predictive models across ethnicities/races could also be further investigated using other types of brain features (in particular multimodal feature sets).

In the same vein, on the behavioral measures side, biases against specific populations in psychometric tools have been a long-standing issue (*59*–*62*). Despite the fact that the behavioral tests used in the HCP and ABCD cohorts are relatively standard tools whose psychometric properties have been previously evaluated, the construct validity (whether the test accurately captures the psychological concept it is intended to measure) of some measures in ethnic minorities cannot be fully guaranteed. In that view, discrepancies in the psychometric data across ethnicities cannot be straightforwardly considered as reflecting biologically difference but should rather be seen as a complicated, entangled result of societal, cultural, and educational factors. Taking an example of a standardized academic achievement test such as SAT (a standardized test for college admissions in the United States), biases against unprivileged ethnic/racial groups have been reported decades ago. First, socioeconomic status and family background including parental education were considered to substantially affect child development and academic test score via factors such as nutrition and out-of-schooling test preparation (*63*, *64*). Inequalities in socioeconomic status and family background across ethnic/racial groups could hence act as a proxy for disparities in academic achievement test. Second, historical segregation of neighborhoods and schools among ethnicities/races has also been discussed as contributing to the academic testing disparities (*65*). In turn, reliance on these standardized testing scores during school admission as gatekeepers might perpetuate inequalities in future life quality and career success across ethnicities/races. Greater care should hence be taken when collecting psychometric data in future initiatives to evaluate their validity in specific ethnicities. Awareness should also be raised to avoid machine learning models acting as new "gatekeepers" from further contributing to structural inequality.

In this study, we have considered and adjusted for multiple variables often reported to be correlated with ethnicity/race. Beyond these variables, further information on family background, neighborhood environment, and accessible resources might be able to help in reducing biases in prediction outcomes. As we have mentioned, family background and neighborhood environment could influence cognitive and academic development and ultimately achievements but may be disparate across ethnic/racial groups. We considered including parental employment in the analyses on the ABCD dataset. However, the related questionnaire only probes for coarse categories by asking whether the parents were working, looking for work, retired, a stay-at-home parent, a student, or others. Nevertheless, it bears mentioning that the ABCD dataset also includes both parent-reported and child-reported ratings on neighborhood safety and crime. These variables are generally not considered in neuroimaging studies but might deserve some attention in future work. Resources that are crucial for the development of children (e.g., youth development units, libraries, and adequate and healthy nutrition) in different

communities could also play an important role in both brain structural and functional development and behavioral measures including cognitive performance and mental health. When predicting psychometric measures that are correlated with certain mental disorders, the health resources that can be accessed by different ethnic/racial groups, especially psychological-medical care services, might be helpful to explain the inequality in prediction results.

Last, our study could also be discussed in the context of the debate around the principle of "fairness through unawareness" in the context of algorithmic fairness. Fairness through unawareness refers to the approach where algorithms do not explicitly interrogate protected variables (ethnicity/race here) in the prediction process. As, in this study, we trained ethnicity/race-specific models to explore the effects of the relative representation in our participant sample on prediction generalizability across subpopulations, it may be seen as a violation of that principle. In the same vein, caution should also be taken in interpreting the patterns learned by ethnicity/race-specific models. In addition to the limited interpretability of machine learning models from a technical standpoint, causal reasoning is precluded by the limitations of studying the treatment effect of ethnicity/race in a counterfactual framework as discussed above. We would here reiterate again that speculating about the causes of observed differences in brain-behavior patterns between two population groups requires taking into account the myriad of factors influencing brain functioning from the gestational period to the time of evaluation, as well as the broad range of sociopsychological factors influencing performance during psychometric testing (such as stereotype threat). We would thus argue against naïve and simplified theorization based on ancestral/genetic differences.

To conclude, we have observed unfair behavioral prediction results when comparing predictive models for WA and AA using a widely adopted training approach in the field. The difference in prediction performance between the two groups was partly related to the dominance of WA in the datasets. More neuroimaging and psychometric data need to be collected from minority ethnic/racial groups, not only within the U.S. population but also from a global perspective. Although the ethnic/racial data composition was important for the fairness of prediction results, it was not able to fully explain the difference in prediction performance. Therefore, future studies are needed to investigate the cross-ethnic validity of possible factors such as data preprocessing and psychometric tools.

MATERIALS AND METHODS

Overview of datasets

Two publicly available datasets were used: the HCP S1200 release (*66*) and the ABCD 2.0.1 release (*67*, *68*). Both datasets contain neuroimaging scans and behavioral measures for individual participants.

HCP participants (N = 1094; age, 22 to 37 years) were healthy and young, including twins and siblings. Family structures were carefully taken care of during latter data splitting and matching (see below). All imaging data were acquired on a customized Siemens 3-T Skyra at Washington University (St. Louis) using a multiband sequence. The structural images were 0.7 mm isotropic. The rs-fMRI data were 2 mm isotropic with Repetition time (TR) = 0.72 s. Two sessions of rs-fMRI data were collected in consecutive days for each participant, and each session consisted of one or two runs. The length of each rs-fMRI scan was 14.4 min (1200 frames). Details of the data collection can be found elsewhere (*23*, *69*). Details about behavioral measures can be found in HCP S1200 Data Dictionary and in (*70*).

ABCD participants (N = 11,875; age, 9 to 11 years) were recruited from 21 sites across the United States (*71*). The imaging data were acquired from multiple 3-T scanner platforms, GE, Philips, and Siemens, with harmonized protocols. The structural images were 1 mm isotropic. Each rs-fMRI run was collected in 2.4-mm isotropic resolution with TR = 0.8 s using a multiband sequence. For each participant, 20 min of rs-fMRI data was acquired in four 5-min runs. Further information about sample selection and recruitment and imaging acquisition can be found elsewhere (*68*, *71*). Details about behavioral measures can be found in (*72*, *73*).

HCP preprocessing and behavioral data

The preprocessing of the HCP neuroimaging data followed previous works (*74*). The MSM-All (*75*)–registered rs-fMRI data were denoised with ICA-FIX (FMRIB's ICA-based X-noiseifier) (*76*) and saved in the CIFTI grayordiante format (*77*). To further reduce the global motion and respirational artifacts (*78*, *79*) and to improve behavioral prediction performance (*20*, *74*), we performed additional regression of global signal (the signal averaged across cortical vertices) and its first temporal derivative. Censoring was performed during global signal regression based on the framewise displacement (FD) (*80*) and the root mean square of voxel-wise differentiated signal (DVARS) (*81*). Mathematically, $\mathrm{DVARS}_i = \sqrt{\langle \Delta I_i(x)^2 \rangle} = \sqrt{\langle [I_i(x) - I_{i-1}(x)]^2 \rangle}$, where $I_i(x)$ is the image intensity at location x of frame i, and the average is taken over all possible x. Specifically, volumes with FD > 0.2 mm or DVARS > 75 were marked as censored frames. One frame before and two frames after these volumes along with the uncensored segments of data lasting fewer than five contiguous volumes were also flagged as censored frames. Regression coefficients were only estimated from the uncensored frames. Rs-fMRI runs with more than half of the frames flagged as censored frames were discarded.

Fifty-eight behavioral variables (table S1) across cognition, personality, and emotion domains were selected (*19*, *74*). Age, gender, FD, DVARS, intracranial volume, years of education, and household income were selected as confounding variables in the behavioral prediction models. These confounding variables were regressed out from both behavioral scores and RSFC during prediction. Of the 1029 participants who passed the motion censoring, 81 participants were excluded because of missing data for behavioral measures or confounding variables, resulting in 948 participants.

ABCD preprocessing and behavioral data

The preprocessing of ABCD neuroimaging data followed (*82*). The structural T1 imaging data were submitted to the ABCD minimal preprocessing pipeline (*83*) and further processed using FreeSurfer 5.3.0 (*84*). The FreeSurfer processing steps generated accurate cortical surface meshes for each individual and registered the individual cortical surface meshes to a common spherical coordinate system (*85*, *86*). A total of 404 participants were removed because they did not pass FreeSurfer recon-all quality control (QC).

The rs-fMRI data were processed by the minimal preprocessing pipeline (*83*) and the following steps: (i) removal of the first X volumes (Siemens and Philips: X = 8; GE DV25: X = 5; GE DV26: X = 16; following the ABCD release notes); (ii) alignment with the T1 images using boundary-based registration (*87*) with FsFast

(http://surfer.nmr.mgh.harvard.edu/fswiki/FsFast); (iii) outlier frame detection (i.e., censoring; see below); (iv) nuisance regression of six motion parameters, global signal, mean white matter signal, mean ventricular signal, and their temporal derivatives (18 regressors in total); (v) interpolation of censored frames using Lomb-Scargle periodogram (*88*); (vi) bandpass filtering (0.009 Hz $\leq f \leq$ 0.08 Hz); and (vii) projection to FreeSurfer fsaverage6 surface space and smoothing using a 6-mm full width at half maximum kernel.

We removed the functional runs with a boundary-based registration cost greater than 0.6. During nuisance regression, the regression coefficients were only estimated from the uncensored frames. Specifically, volumes with FD > 0.3 mm or DVARS > 50, along with one volume before and two volumes after, were marked as censored frames. The FD threshold used for ABCD was chosen following (*82*) to achieve the balance between adequate motion censoring and a large sample size. The DVARS thresholds were selected so that the number of censored frames due to DVARS was roughly the same as the number of frames due to FD. Uncensored segments of data containing fewer than five contiguous volumes were also censored. Rs-fMRI runs with more than half of the frames flagged as censored frames were discarded. After censoring, the participants with less than 4-min rs-fMRI data were excluded. In total, 4457 participants were discarded following rs-fMRI QC. Furthermore, 11 participants were excluded because the data were collected on the problematic Philips scanners (https://github.com/ABCD-STUDY/fMRI-cleanup).

Different from the preprocessing approach used in the HCP dataset, ICA-FIX was not adopted in the ABCD dataset. A potential limitation of ICA-FIX for the current study could be that the signal-versus-noise classifier was also trained in the HCP dataset (*76*), which was again dominated by WA. However, we acknowledge that the current preprocessing strategies for the ABCD dataset were following our previous studies (*19*, *74*, *82*) and might not be optimal.

We examined 36 behavioral measures (*82*) from all available neurocognitive (*72*) and mental health (*73*) assessments, except the Kiddie Schedule for Affective Disorders and Schizophrenia (KSADS-5) and Cash Choice Task because these two measures contained binary or ternary values that were not suitable for regression model. These 36 behavioral measures are summarized in table S2. Age, gender, FD, DVARS, intracranial volume, and parental education were considered as confounding variables in the behavioral prediction models. Among the 5809 participants who passed both recon-all and rs-fMRI QC, 458 participants were excluded because of missing data for behavioral or confounding variables, yielding 5351 participants across 19 sites for further analyses.

Functional connectivity computation

RSFC was computed across 419 regions of interest (ROIs) (Fig. 1, C and D) using Pearson's correlation. The 419 ROIs consisted of 400 cortical parcels (https://github.com/ThomasYeoLab/CBIG/tree/master/stable_projects/brain_parcellation/Schaefer2018_LocalGlobal) (*56*) and 19 subcortical regions. For the HCP dataset, to be consistent with the space of fMRI time series, these 19 subcortical regions were defined on the basis of grayordinate data structure. For the ABCD dataset, the 19 subcortical ROIs were obtained in participant-specific volumetric space defined by FreeSurfer (*89*). Censored frames were ignored in the RSFC calculation. For each participant, the correlation matrix was computed for each run, Fisher *z*-transformed, and then averaged across runs and sessions, yielding one final 419 × 419 RSFC matrix for each participant.

Subpopulation matching and data split

Differences in prediction accuracy between WA and AA, if any, could be related to confounding effects of variables such as head size (and thus brain size). To limit the influence of confounding factors as much as possible, Hungarian matching was performed to select demographically and behaviorally matched AA-WA pairs, which were used for the test phase of cross-validation in behavioral prediction. Hungarian matching was initially proposed to solve the assignment problem (*90*, *91*). A simple example of assignment problem can be considered: Three workers are supposed to be assigned to three jobs separately. Each worker asks for different payments for completing different jobs, which generates a 3 × 3 matrix where element (i, j) of this matrix represents the cost of assigning worker i to job j. The goal is to assign each worker to a job with the lowest total cost. In this study, we treated WA and AA as the workers and jobs in the previous example and considered the differences in behavioral scores and confounding factors as the cost.

Because of the single- versus multisite difference between the two datasets, procedures for matching and data split were different. For the HCP dataset, the participants can be randomly split into folds, and the matching can be achieved within each fold, as long as the family structure was taken care of. To obtain generalizable results, the random split can be performed multiple times with different random seeds. However, for the ABCD dataset, the matching was performed within each site. Given the various site sizes, the 19 sites cannot be combined into training and test folds with random repetitions. Hence, we first combined the 19 sites into 10 folds that had a similar number of matched pairs of AA and WA. Then, each time, the prediction model was trained on 7 of the 10 folds and tested on the other 3 folds, yielding 120 variations of training-test split for generalizability purposes.

The 948 HCP participants included 721 WA (62 Hispanic + 659 non-Hispanic), 129 AA, 59 Asian/native Hawaiian/other Pacific Islander participants, 2 American Indians/Alaskan natives, 22 participants with mixed ethnicities/races, and 15 participants with unknown or nonreported ethnic/racial information (Fig. 1A). For each behavior, Hungarian matching was used to select the matched AA-WA pairs for the comparison of prediction accuracies. According to an initial matching step, 28 AA participants exhibited consistently high matching cost across behaviors, where the matching cost was calculated as the summed differences in age, gender, FD, DVARS, and behavioral scores. Hence, they were excluded from the matching procedure and grouped together with other ethnic/racial subpopulations (Fig. 2A, yellow color). Post hoc inspection observed higher proportion of females, more poorly educated, and low-income participants within these 28 participants, compared to the entire AA sample involved in this study. The remaining 101 AA (blue color) were randomly split into 10 folds for cross-validation in behavioral prediction. The same number of WA (green color) was randomly selected from the total 721 WA, repeated by 10,000 iterations. Within each iteration, we calculated the AA-WA matching cost as mentioned above for each fold. The final matched WA were from the iteration with the lowest maximal cost across folds. Additional matching for intracranial volume, education, and income was not possible because it would have greatly decreased the selected AA sample size. For each behavior, the whole procedure was randomly repeated multiple times until at least 40 different AA splits can be obtained with matched WA, because a single 10-fold cross-validation might be sensitive to the particular split of the data

into folds (*92*). For each random split of AA, the 620 unmatched WA, along with the 28 hard-to-match AA and participants from other ethnic/racial groups, were further randomly split into 10 folds and then were combined with the 10 folds of matched AA-WA pairs. We ensured that participants from the same family were not split across folds among all steps. Differences in the matching confounds and behavioral distributions between the selected AA-WA pairs were evaluated using paired sample *t* test. Multiple comparisons were corrected on the basis of FDR < 0.05 across all behaviors, confounds, and data splits. For the following seven behaviors, matched WA could not be found for enough (i.e., 40) AA splits: fluid intelligence (Penn Matrix Test), vocabulary (picture matching), delay discounting, story comprehension, relational processing, working memory (N-back), and life satisfaction. These behavioral measures were excluded from further analyses. No significant difference was found for the remaining behavioral measures after correction (fig. S1).

The 5351 ABCD participants included 2999 WA (non-Hispanic), 635 AA, 1053 Hispanic participants, 110 Asian participants, and 554 participants in other ethnic/racial groups, spread across 19 sites (Fig. 1B; purple color in Fig. 2B). For each behavior, iterative Hungarian matching was performed within each site to obtain matched AA-WA pairs based on the summed differences in age, gender, FD, DVARS, intracranial volume, parental education, and the behavioral scores. Starting from all AA of a specific site, each iteration excluded the AA that could not be matched with any WA and the AA with the highest matching cost. The iteration stopped when it reached 100 rounds or the decrease of matching cost was less than 5% of the cost in the previous iteration, yielding the matched AA and WA for each site. The thresholds for stopping the iterations were chosen not only to obtain matched AA-WA pairs with no significant difference in demographical and behavioral distributions but also to keep as many participants as possible. The matched AA and WA were represented by blue and green colors in Fig. 2B, respectively, while the unmatched AA, WA, and participants in other race groups were indicated by yellow color. In summary, the number of matched pairs of AA and WA ranged from 192 to 301 across behaviors. The 19 sites, including all ethnic/racial groups, were further merged into 10 folds to achieve more similar number of matched AA-WA pairs across the 10 folds. Because of the site constraints, data splitting could not be randomly repeated as for the HCP dataset. Instead, we selected any 7 of the 10 folds to train the behavioral model, and the remaining 3 folds were used to test the model. Differences in confounds and behavioral distributions between the matched AA and WA were evaluated using paired sample *t* test (FDR-corrected across all behaviors, confounds, and data splits). No significant difference was found for all 36 behaviors after correction (fig. S2).

Kernel ridge regression

Each behavioral measure from both datasets was predicted using kernel ridge regression (*93*) separately. We used this approach because it can effectively predict behavioral measures while enjoying low computational cost. Suppose y_s and y_i denote the behavioral measure (e.g., episodic memory) of test participant *s* and training participant *i*, respectively. Let c_s and c_i denote the vectorized RSFC (lower triangular entries of the RSFC matrices) of test participant *s* and training participant *i*, respectively. Then, roughly speaking, kernel regression would predict y_s as the weighted average of the behavioral measures of all training participants, i.e., $\hat{y}_s \approx \sum_{i \in \text{training set}} \text{Similarity}(c_s, c_i)\, y_i$. Here, Similarity($c_s$, c_i) was defined by Pearson's correlation between the vectorized RSFC of the test participant and the *i*th training participant. Therefore, successful prediction would indicate that participants with more similar RSFC have similar behavioral scores. To reduce overfitting, an l_2 regularization term was included. More details can be found in Supplementary Methods.

For the HCP dataset, we performed 10-fold nested cross-validation preserving family structure. For each test fold, the kernel regression parameters were estimated from all ethnic/racial groups in the nine training folds. Tenfold cross-validation was, in turn, performed on the nine training folds with different l_2 regularization parameter λ to optimally select the value of λ. The estimated parameters from the training folds were then used to predict the behavior of the participants in the test fold. Because a single 10-fold cross-validation might be sensitive to the particular split of the data into folds (*33*), the above 10-fold cross-validation was repeated 40 times (see the "Subpopulation matching and data split" section). Confounding variables of age, gender, FD, DVARS, intracranial volume, education, and household income were regressed out from both behavioral and RSFC data. To investigate the effects of confound regression on the model biases, we also repeated the main analysis without regressing any confounding variables.

For the ABCD dataset, all participants were split into 10 folds (see the "Subpopulation matching and data split" section). The kernel regression parameters were estimated from any seven folds and applied on the remaining three folds for testing, yielding 120 training-test combinations (*82*). The 10-fold cross-validation was performed on the 7 training folds to select the optimal regulation parameter λ. Confounding variables of age, gender, FD, DVARS, intracranial volume, and parental education were regressed from both behavioral and RSFC data. Same as for the HCP dataset, we also repeated the main analysis without confound regression. Household income was not included because of the large number of missing values (93 of the total 635 AA participants refused to answer or with unknown income). To ensure that the results were not specific to kernel ridge regression models, we also used linear ridge regression as an auxiliary method for both datasets. The training and test procedures were the same as those used for kernel ridge regression. The selected optimal hyperparameters of these two methods are listed in table S3 for each behavioral measure.

Two accuracy metrics were considered: predictive COD and Pearson's correlation. For each test fold, the predictive COD of AA was defined as $1 - \text{SSE}_{\text{AA}}/\text{SST}$, where $\text{SSE}_{\text{AA}} = \sum_{i \in \text{test AA}} (y_i - \hat{y}_i)^2 / N_{\text{test AA}}$ (y_i and $\hat{y}_i$ are the original behavioral score and predicted score of *i*th test AA participant, respectively; $N_{\text{test AA}}$ is the number of AA in test set), i.e., the MSE. The denominator $\text{SST} = \sum_{j \in \text{train AA\&WA}} (y_j - \text{mean}(y_{\text{train AA\&WA}}))^2 / N_{\text{train AA\&WA}}$ represented the total behavioral variance learned from the training set. The predictive COD of WA was defined as $1 - \text{SSE}_{\text{WA}}/\text{SST}$, where $\text{SSE}_{\text{WA}} = \sum_{i \in \text{test WA}} (y_i - \hat{y}_i)^2 / N_{\text{test WA}}$ and SST was the same as AA because the total variance was not assumed here to be group specific. Pearson's correlation was also calculated separately for each test fold. For the HCP dataset, the predictive COD or Pearson's correlation was averaged across 10 folds for each data split, yielding 40 accuracy values. For the ABCD dataset, the 120 accuracy values corresponding to 120 data splits were not averaged but directly shown in the boxplots in Results.

AA versus WA accuracy difference

For each dataset and each accuracy metric, we determined behaviors as predictable or not based on two criteria: (i) the accuracy across all

test participants including AA, WA, and other ethnicity/race that survived the multilevel block permutation test (*94*) by shuffling the predicted behavioral scores 1000 times (FDR-corrected across behaviors); (ii) the average accuracies across data splits were positive in either AA or WA. For each predictable behavior, the accuracy difference between matched AA and WA was evaluated by a permutation test, where the null distribution was built by recalculating accuracies with shuffling the group labels 1000 times. Multiple comparisons were controlled with FDR < 0.05.

Influence of training population

To explore the effects of training population, we trained the kernel ridge regression model specifically on only-AA or only-WA subsamples separately. Concretely, we selected all AA in the training folds. Within each site of selected training AA, we randomly selected the same number of WA. Note that for some sites, the total number of WA was fewer than AA; hence, random AA were excluded to match the number of WA. The prediction model was then trained on the selected AA or the selected WA or both and tested on matched AA and WA, in the same way as when the model was trained on the full datasets.

Brain-behavior association pattern

As stated in (*34*), the model parameters learned by kernel ridge regression cannot be interpreted directly as the importance of the corresponding pair of ROIs. Using the inversion method provided in (*34*), the importance of each edge, referred to as model-learned brain-behavior association, can be calculated as covariance($\mathrm{RSFC}_{\mathrm{train}}^{pq}$, $\hat{y}_{\mathrm{train}}$), where $\mathrm{RSFC}_{\mathrm{train}}^{pq}$ is the demeaned RSFC between pth and qth ROIs of all training participants, and $\hat{y}_{\mathrm{train}}$ is the predicted behavioral scores of training participants. Similarly, the true brain-behavior association pattern can be defined separately for AA and WA as covariance($\mathrm{RSFC}_{\mathrm{test\ AA}}^{pq}$, $y_{\mathrm{test\ AA}}$) and covariance($\mathrm{RSFC}_{\mathrm{test\ WA}}^{pq}$, $y_{\mathrm{test\ WA}}$). Note that the true brain-behavior association was computed on the basis of the original behavioral scores instead of predicted scores. Pearson's correlation between model-learned and true brain-behavior association was then calculated as the similarity between the two measures for AA and WA separately.

SUPPLEMENTARY MATERIALS

Supplementary material for this article is available at https://science.org/doi/10.1126/sciadv.abj1812

View/request a protocol for this paper from *Bio-protocol*.

REFERENCES AND NOTES

1. H. Fröhlich, R. Balling, N. Beerenwinkel, O. Kohlbacher, S. Kumar, T. Lengauer, M. H. Maathuis, Y. Moreau, S. A. Murphy, T. M. Przytycka, M. Rebhan, H. Röst, A. Schuppert, M. Schwab, R. Spang, D. Stekhoven, J. Sun, A. Weber, D. Ziemek, B. Zupan, From hype to reality: Data science enabling personalized medicine. *BMC Med.* **16**, 150 (2018).
2. D. Cirillo, A. Valencia, Big data analytics for personalized medicine. *Curr. Opin. Biotechnol.* **58**, 161–167 (2019).
3. Z. Ahmed, K. Mohamed, S. Zeeshan, X. Q. Dong, Artificial intelligence with multi-functional machine learning platform development for better healthcare and precision medicine. *Database* **2020**, baaa010 (2020).
4. S. I. Lee, S. Celik, B. A. Logsdon, S. M. Lundberg, T. J. Martins, V. G. Oehler, E. H. Estey, C. P. Miller, S. Chien, J. Dai, A. Saxena, C. A. Blau, P. S. Becker, A machine learning approach to integrate big data for precision medicine in acute myeloid leukemia. *Nat. Commun.* **9**, 42 (2018).
5. V. Gulshan, L. Peng, M. Coram, M. C. Stumpe, D. Wu, A. Narayanaswamy, S. Venugopalan, K. Widner, T. Madams, J. Cuadros, R. Kim, R. Raman, P. C. Nelson, J. L. Mega, D. R. Webster, Development and validation of a deep learning algorithm for detection of diabetic retinopathy in retinal fundus photographs. *J. Am. Med. Assoc.* **316**, 2402–2410 (2016).
6. I. Shomorony, E. T. Cirulli, L. Huang, L. A. Napier, R. R. Heister, M. Hicks, I. V. Cohen, H. C. Yu, C. L. Swisher, N. M. Schenker-Ahmed, W. Li, K. E. Nelson, P. Brar, A. M. Kahn, T. D. Spector, C. T. Caskey, J. C. Venter, D. S. Karow, E. F. Kirkness, N. Shah, An unsupervised learning approach to identify novel signatures of health and disease from multimodal data. *Genome Med.* **12**, 7 (2020).
7. A. Chouldechova, E. Putnam-Hornstein, S. Dworak-Peck, D. Benavides-Prado, O. Fialko, R. Vaithianathan, S. A. Friedler, C. Wilson, A case study of algorithm-assisted decision making in child maltreatment hotline screening decisions. *Proc. Mach. Learn. Res.* **81**, 134–148 (2018).
8. A. R. Martin, M. Kanai, Y. Kamatani, Y. Okada, B. M. Neale, M. J. Daly, Clinical use of current polygenic risk scores may exacerbate health disparities. *Nat. Genet.* **51**, 584–591 (2019).
9. Z. Obermeyer, B. Powers, C. Vogeli, S. Mullainathan, Dissecting racial bias in an algorithm used to manage the health of populations. *Science* **366**, 447–453 (2019).
10. J. Buolamwini, T. Gebru, Gender shades: Intersectional accuracy disparities in commercial gender classification. *Proc. Mach. Learn. Res.* **81**, 77–91 (2018).
11. A. Chouldechova, A. Roth, The frontiers of fairness in machine learning. arXiv:1810.08810 (2018).
12. S. R. Pfohl, A. Foryciarz, N. H. Shah, An empirical characterization of fair machine learning for clinical risk prediction. *J. Biomed. Inform.* **113**, 103621 (2020).
13. R. Zemel, Y. Wu, K. Swersky, T. Pitassi, C. Dwork, Learning fair representations. *Proc. Mach. Learn. Res.* **28**, 325–333 (2013).
14. M. Hardt, E. Price, N. Srebro, Equality of opportunity in supervised learning. *Adv. Neural Inf. Process. Syst.*, 3323–3331 (2016).
15. A. Rajkomar, M. Hardt, M. D. Howell, G. Corrado, M. H. Chin, Ensuring fairness in machine learning to advance health equity. *Ann. Intern. Med.* **169**, 866–872 (2018).
16. G. Pleiss, M. Raghavan, F. Wu, J. Kleinberg, K. Q. Weinberger, On fairness and calibration. arXiv:1709.02012 (2017).
17. A. Zink, S. Rose, Fair regression for health care spending. *Biometrics* **76**, 973–982 (2020).
18. E. S. Finn, X. Shen, D. Scheinost, M. D. Rosenberg, J. Huang, M. M. Chun, X. Papademetris, R. Todd Constable, Functional connectome fingerprinting: Identifying individuals using patterns of brain connectivity. *Nat. Neurosci.* **18**, 1664–1671 (2015).
19. R. Kong, J. Li, C. Orban, M. R. Sabuncu, H. Liu, A. Schaefer, N. Sun, X.-N. Zuo, A. J. Holmes, S. B. Eickhoff, B. T. T. Yeo, Spatial topography of individual-specific cortical networks predicts human cognition, personality, and emotion. *Cereb. Cortex* **29**, 2533–2551 (2019).
20. A. S. Greene, S. Gao, D. Scheinost, R. T. Constable, Task-induced brain state manipulation improves prediction of individual traits. *Nat. Commun.* **9**, 2807 (2018).
21. E. S. Finn, P. A. Bandettini, Movie-watching outperforms rest for functional connectivity-based prediction of behavior. *NeuroImage* **235**, 117963 (2021).
22. J. Wu, S. B. Eickhoff, F. Hoffstaedter, K. R. Patil, H. Schwender, S. Genon, A connectivity-based psychometric prediction framework for brain-behavior relationship studies. *bioRxiv* 2020.01.15.907642 , (2020).
23. D. C. Van Essen, K. Ugurbil, E. Auerbach, D. Barch, T. E. J. Behrens, R. Bucholz, A. Chang, L. Chen, M. Corbetta, S. W. Curtiss, S. Della Penna, D. Feinberg, M. F. Glasser, N. Harel, A. C. Heath, L. Larson-Prior, D. Marcus, G. Michalareas, S. Moeller, J. Xu, E. Yacoub; WU-Minn HCP Consortium, The human connectome project: A data acquisition perspective. *NeuroImage* **62**, 2222–2231 (2012).
24. C. Sudlow, J. Gallacher, N. Allen, V. Beral, P. Burton, J. Danesh, P. Downey, P. Elliott, J. Green, M. Landray, B. Liu, P. Matthews, G. Ong, J. Pell, A. Silman, A. Young, T. Sprosen, T. Peakman, R. Collins, UK biobank: An open access resource for identifying the causes of a wide range of complex diseases of middle and old age. *PLoS Med.* **12**, e1001779 (2015).
25. W. Yan, V. Calhoun, M. Song, Y. Cui, H. Yan, S. Liu, L. Fan, N. Zuo, Z. Yang, K. Xu, J. Yan, L. Lv, J. Chen, Y. Chen, H. Guo, P. Li, L. Lu, P. Wan, H. Wang, H. Wang, Y. Yang, H. Zhang, D. Zhang, T. Jiang, J. Sui, Discriminating schizophrenia using recurrent neural network applied on time courses of multi-site FMRI data. *EBioMed.* **47**, 543–552 (2019).
26. K. G. Noble, M. E. Wolmetz, L. G. Ochs, M. J. Farah, B. D. McCandliss, Brain-behavior relationships in reading acquisition are modulated by socioeconomic factors. *Dev. Sci.* **9**, 642–654 (2006).
27. K. G. Noble, S. M. Houston, N. H. Brito, H. Bartsch, E. Kan, J. M. Kuperman, N. Akshoomoff, D. G. Amaral, C. S. Bloss, O. Libiger, N. J. Schork, S. S. Murray, B. J. Casey, L. Chang, T. M. Ernst, J. A. Frazier, J. R. Gruen, D. N. Kennedy, P. Van Zijl, S. Mostofsky, W. E. Kaufmann, T. Kenet, A. M. Dale, T. L. Jernigan, E. R. Sowell, Family income, parental education and brain structure in children and adolescents. *Nat. Neurosci.* **18**, 773–778 (2015).
28. U. A. Tooley, A. P. MacKey, R. Ciric, K. Ruparel, T. M. Moore, R. C. Gur, R. E. Gur, T. D. Satterthwaite, D. S. Bassett, Associations between neighborhood SES and functional brain network development. *Cereb. Cortex* **30**, 1–19 (2020).
29. J. Komiyama, A. Takeda, J. Honda, and H. Shimao, Nonconvex optimization for regression with fairness constraints, in *Proceedings of the 35th International Conference on Machine Learning* (2018), pp. 80:2737–2746.
30. R. Berk, H. Heidari, S. Jabbari, M. Joseph, M. Kearns, J. Morgenstern, S. Neel, A. Roth, A convex framework for fair regression. arXiv:1706.02409 (2017).

31. A. Agarwal, M. Dudík, Z. S. Wu, Fair regression: Quantitative definitions and reduction-based algorithms, in *Proceedings of the 36th International Conference on Machine Learning* (2019), pp. 97:120–129.
32. T. He, R. Kong, A. J. Holmes, M. Nguyen, M. R. Sabuncu, S. B. Eickhoff, D. Bzdok, J. Feng, B. T. T. Yeo, Deep neural networks and kernel regression achieve comparable accuracies for functional connectivity prediction of behavior and demographics. *NeuroImage* **206**, 116276 (2020).
33. D. Scheinost, S. Noble, C. Horien, A. S. Greene, E. M. Lake, M. Salehi, S. Gao, X. Shen, D. O'Connor, D. S. Barron, S. W. Yip, M. D. Rosenberg, R. T. Constable, Ten simple rules for predictive modeling of individual differences in neuroimaging. *NeuroImage* **193**, 35–45 (2019).
34. S. Haufe, F. Meinecke, K. Görgen, S. Dähne, J. D. Haynes, B. Blankertz, F. Bießmann, On the interpretation of weight vectors of linear models in multivariate neuroimaging. *NeuroImage* **87**, 96–110 (2014).
35. R. E. Beaty, Y. N. Kenett, A. P. Christensen, M. D. Rosenberg, M. Benedek, Q. Chen, A. Fink, J. Qiu, T. R. Kwapil, M. J. Kane, P. J. Silvia, Robust prediction of individual creative ability from brain functional connectivity. *Proc. Natl. Acad. Sci.* **115**, 1087–1092 (2018).
36. U. Pervaiz, D. Vidaurre, M. W. Woolrich, S. M. Smith, Optimising network modelling methods for fMRI. *NeuroImage* **211**, 116604 (2020).
37. B. Cai, G. Zhang, A. Zhang, L. Xiao, W. Hu, J. M. Stephen, T. W. Wilson, V. D. Calhoun, Y. P. Wang, Functional connectome fingerprinting: Identifying individuals and predicting cognitive function via deep learning. arXiv:2006.09928 (2020).
38. S. B. Eickhoff, R. Langner, Neuroimaging-based prediction of mental traits: Road to utopia or Orwell? *PLoS Biol.* **17**, e3000497 (2019).
39. S. L. Mansour, Y. Tian, B. T. T. Yeo, V. Cropley, A. Zalesky, High-resolution connectomic fingerprints: Mapping neural identity and behavior. *NeuroImage* **229**, 117695 (2021).
40. N. Pearce, Analysis of matched case-control studies. *BMJ* **352**, i969 (2016).
41. P. R. Rosenbaum, D. B. Rubin, The central role of the propensity score in observational studies for causal effects. *Biometrika* **70**, 41–55 (1983).
42. M. Blackwell, S. Iacus, G. King, G. Porro, cem: Coarsened exact matching in Stata. *Stata J.* **9**, 524–546 (2009).
43. L. J. Krivo, R. L. Kaufman, Housing and wealth inequality: Racial-ethnic differences in home equity in the United States. *Demography* **41**, 585–605 (2004).
44. A. Gamoran, R. N. L. Turley, A. Turner, R. Fish, Differences between Hispanic and non-Hispanic families in social capital and child development: First-year findings from an experimental study. *Res. Soc. Stratif. Mobil.* **30**, 97–112 (2012).
45. D. O'Connor, E. M. R. Lake, D. Scheinost, R. T. Constable, Resample aggregating improves the generalizability of connectome predictive modeling. *NeuroImage* **236**, 118044 (2021).
46. E. Alemán Jr., Situating texas school finance policy in a CRT framework: How 'substantially equal' yields racial inequity. *Educ. Adm. Q.* **43**, 525–558 (2007).
47. L. Darling-Hammond, Inequality and school resources: What it will take to close the oppotunity gap, in *Closing the Opportunity Gap: What America Must do to Give Every Child an Even Chance* (Oxford Univ. Press, 2013), p. 77.
48. V. J. Roscigno, Race and the reproduction of educational disadvantage. *Soc. Forces* **76**, 1033–1060 (1998).
49. M. Romero, E. Margolis, *The Blackwell Companion to Social Inequalities* (John Wiley & Sons, 2008).
50. M. J. Farah, Socioeconomic status and the brain: Prospects for neuroscience-informed policy. *Nat. Neurosci.* **19**, 428–438 (2018).
51. D.-S. Murray, L. Richardson, R. Tesfaye, D. Nadin, C. Kelly, P. Greenwood, Black in neuro, beyond one week. *J. Neurosci.* **41**, 2314–2317 (2021).
52. T. J. VanderWeele, W. R. Robinson, On the causal interpretation of race in regressions adjusting for confounding and mediating variables. *Epidemiology* **25**, 473–484 (2014).
53. I. Kohler-Hausmann, Eddie Murphy and the Dangers of Counterfactual Causal Thinking About Detecting Racial Discrimination. *SSRN Electron. J.* (2019), doi: 10.2139/SSRN.3050650.
54. P. Liang, L. Shi, N. Chen, Y. Luo, X. Wang, K. Liu, V. C. T. Mok, W. C. W. Chu, D. Wang, K. Li, Construction of brain atlases based on a multi-center MRI dataset of 2020 Chinese adults. *Sci. Rep.* **5**, 18216 (2016).
55. G. Yang, S. Zhou, J. Bozek, H. M. Dong, M. Han, X. N. Zuo, H. Liu, J. H. Gao, Sample sizes and population differences in brain template construction. *NeuroImage* **206**, 116318 (2020).
56. A. Schaefer, R. Kong, E. M. Gordon, T. O. Laumann, X.-N. Zuo, A. J. Holmes, S. B. Eickhoff, B. T. T. Yeo, Local-global parcellation of the human cerebral cortex from intrinsic functional connectivity MRI. *Cereb. Cortex* **28**, 3095–3114 (2018).
57. R. Kong, Q. Yang, E. Gordon, A. Xue, X. Yan, C. Orban, X.-N. Zuo, N. Spreng, T. Ge, A. Holmes, S. Eickhoff, B. T. T. Yeo, Individual-specific areal-level parcellations improve functional connectivity prediction of behavior. *Cereb. Cortex* **31**, 4477–4500 (2021).
58. M. Salehi, A. S. Greene, A. Karbasi, X. Shen, D. Scheinost, R. T. Constable, There is no single functional atlas even for a single individual: Functional parcel definitions change with task. *NeuroImage* **208**, 116366 (2020).
59. S. J. Gould, *The Mismeasure of Man* (W. W. Norton & Company, 1996).
60. J. B. Ritsher, E. L. Struening, F. Hellman, M. Guardino, Internal validity of an anxiety disorder screening instrument across five ethnic groups. *Psychiatry Res.* **111**, 199, 213 (2002).
61. C. M. Berry, M. A. Clark, T. K. McClure, Racial/ethnic differences in the criterion-related validity of cognitive ability tests: A qualitative and quantitative review. *J. Appl. Psychol.* **96**, 881–906 (2011).
62. M. M. Elbulok-Charcape, L. A. Rabin, A. T. Spadaccini, W. B. Barr, Trends in the neuropsychological assessment of ethnic/racial minorities: A survey of clinical neuropsychologists in the United States and Canada. *Cult. Divers. Ethn. Minor. Psychol.* **20**, 353–361 (2014).
63. E. Dixon-Román, H. Everson, J. McArdle, Race, poverty and SAT scores: Modeling the influences of family income on black and white high school students' SAT performance. *Teach. Coll. Rec.* **115**, 1–33 (2013).
64. C. Buchmann, D. J. Condron, V. J. Roscigno, Shadow education, American style: Test preparation, the SAT and college enrollment. *Soc. Forces* **89**, 435–461 (2010).
65. M. Knoester, W. Au, Standardized testing and school segregation: Like tinder for fire? *Race Ethn. Educ.* **20**, 1–14 (2017).
66. D. C. Van Essen, S. M. Smith, D. M. Barch, T. E. J. Behrens, E. Yacoub, K. Ugurbil, The WU-minn human connectome project: An overview. *NeuroImage* **80**, 62–79 (2013).
67. N. D. Volkow, G. F. Koob, R. T. Croyle, D. W. Bianchi, J. A. Gordon, W. J. Koroshetz, E. J. Pérez-Stable, W. T. Riley, M. H. Bloch, K. Conway, B. G. Deeds, G. J. Dowling, S. Grant, K. D. Howlett, J. A. Matochik, G. D. Morgan, M. M. Murray, A. Noronha, C. Y. Spong, E. M. Wargo, K. R. Warren, S. R. B. Weiss, The conception of the ABCD study: From substance use to a broad NIH collaboration. *Dev. Cogn. Neurosci.* **32**, 4–7 (2018).
68. H. Garavan, H. Bartsch, K. Conway, A. Decastro, R. Z. Goldstein, S. Heeringa, T. Jernigan, A. Potter, W. Thompson, D. Zahs, Recruiting the ABCD sample: Design considerations and procedures. *Dev. Cogn. Neurosci.* **32**, 16–22 (2018).
69. S. M. Smith, C. F. Beckmann, J. Andersson, E. J. Auerbach, J. Bijsterbosch, G. Douaud, E. Duff, D. A. Feinberg, L. Griffanti, M. P. Harms, M. Kelly, T. Laumann, K. L. Miller, S. Moeller, S. Petersen, J. Power, G. Salimi-Khorshidi, A. Z. Snyder, A. T. Vu, M. W. Woolrich, J. Xu, E. Yacoub, K. Uğurbil, D. van Essen, M. F. Glasser; WU-Minn HCP Consortium, Resting-state fMRI in the human connectome project. *NeuroImage* **80**, 144–168 (2013).
70. D. M. Barch, G. C. Burgess, M. P. Harms, S. E. Petersen, B. L. Schlaggar, M. Corbetta, M. F. Glasser, S. Curtiss, S. Dixit, C. Feldt, D. Nolan, E. Bryant, T. Hartley, O. Footer, J. M. Bjork, R. Poldrack, S. Smith, H. Johansen-Berg, A. Z. Snyder, D. van Essen; WU-Minn HCP Consortium, Function in the human connectome: Task-fMRI and individual differences in behavior. *NeuroImage* **80**, 169–189 (2013).
71. B. J. Casey, T. Cannonier, M. I. Conley, A. O. Cohen, D. M. Barch, M. M. Heitzeg, M. E. Soules, T. Teslovich, D. V. Dellarco, H. Garavan, C. A. Orr, T. D. Wager, M. T. Banich, N. K. Speer, M. T. Sutherland, M. C. Riedel, A. S. Dick, J. M. Bjork, K. M. Thomas, B. Chaarani, M. H. Mejia, D. J. Hagler Jr., M. D. Cornejo, C. S. Sicat, M. P. Harms, N. U. F. Dosenbach, M. Rosenberg, E. Earl, H. Bartsch, R. Watts, J. R. Polimeni, J. M. Kuperman, D. A. Fair, A. M. Dale; ABCD Imaging Acquisition Workgroup, The Adolescent Brain Cognitive Development (ABCD) study: Imaging acquisition across 21 sites. *Dev. Cogn. Neurosci.* **32**, 43–54 (2018).
72. M. Luciana, J. M. Bjork, B. J. Nagel, D. M. Barch, R. Gonzalez, S. J. Nixon, M. T. Banich, Adolescent neurocognitive development and impacts of substance use: Overview of the Adolescent Brain Cognitive Development (ABCD) baseline neurocognition battery. *Dev. Cogn. Neurosci.* **32**, 67–79 (2018).
73. D. M. Barch, M. D. Albaugh, S. Avenevoli, L. Chang, D. B. Clark, M. D. Glantz, J. J. Hudziak, T. L. Jernigan, S. F. Tapert, D. Yurgelun-Todd, N. Alia-Klein, A. S. Potter, M. P. Paulus, D. Prouty, R. A. Zucker, K. J. Sher, Demographic, physical and mental health assessments in the adolescent brain and cognitive development study: Rationale and description. *Dev. Cogn. Neurosci.* **32**, 55–66 (2018).
74. J. Li, R. Kong, R. Liégeois, C. Orban, Y. Tan, N. Sun, A. J. Holmes, M. R. Sabuncu, T. Ge, B. T. T. Yeo, Global signal regression strengthens association between resting-state functional connectivity and behavior. *NeuroImage* **196**, 126–141 (2019).
75. E. C. Robinson, S. Jbabdi, J. Andersson, S. Smith, M. F. Glasser, D. C. Van Essen, G. Burgess, M. P. Harms, D. M. Barch, M. Jenkinson, Multimodal surface matching: Fast and generalisable cortical registration using discrete optimisation, in *International Conference on Information Processing in Medical Imaging* (2013), LNCS, vol. 7917, pp. 475–486.
76. G. Salimi-Khorshidi, G. Douaud, C. F. Beckmann, M. F. Glasser, L. Griffanti, S. M. Smith, Automatic denoising of functional MRI data: Combining independent component analysis and hierarchical fusion of classifiers. *NeuroImage* **90**, 449–468 (2014).
77. M. F. Glasser, S. N. Sotiropoulos, J. A. Wilson, T. S. Coalson, B. Fischl, J. L. Andersson, J. Xu, S. Jbabdi, M. Webster, J. R. Polimeni, D. C. Van Essen, M. Jenkinson; WU-Minn HCP Consortium, The minimal preprocessing pipelines for the Human Connectome Project. *NeuroImage* **80**, 105–124 (2013).
78. G. C. Burgess, S. Kandala, D. Nolan, T. O. Laumann, J. Power, B. Adeyemo, M. P. Harms, S. E. Petersen, D. M. Barch, Evaluation of denoising strategies to address motion-correlated artifacts in resting-state functional magnetic resonance imaging data from the human connectome project. *Brain Connect.* **6**, 669–680 (2016).

79. J. S. Siegel, A. Mitra, T. O. Laumann, B. A. Seitzman, M. Raichle, M. Corbetta, A. Z. Snyder, Data quality influences observed links between functional connectivity and behavior. *Cereb. Cortex* **27**, 4492–4502 (2017).
80. M. Jenkinson, P. Bannister, M. Brady, S. Smith, Improved optimization for the robust and accurate linear registration and motion correction of brain images. *NeuroImage* **17**, 825–841 (2002).
81. J. D. Power, K. A. Barnes, A. Z. Snyder, B. L. Schlaggar, S. E. Petersen, Spurious but systematic correlations in functional connectivity MRI networks arise from subject motion. *NeuroImage* **59**, 2142–2154 (2012).
82. J. Chen, A. Tam, V. Kebets, C. Orban, L. Q. R. Ooi, S. Marek, N. Dosenbach, S. Eickhoff, D. Bzdok, A. Holmes, B. T. Thomas Yeo, Shared and unique brain network features predict cognition, personality and mental health in childhood. *bioRxiv*, 2020.06.24.168724 (2020).
83. D. J. Hagler Jr., S. N. Hatton, M. D. Cornejo, C. Makowski, D. A. Fair, A. S. Dick, M. T. Sutherland, B. J. Casey, D. M. Barch, M. P. Harms, R. Watts, J. M. Bjork, H. P. Garavan, L. Hilmer, C. J. Pung, C. S. Sicat, J. Kuperman, H. Bartsch, F. Xue, M. M. Heitzeg, A. R. Laird, T. T. Trinh, R. Gonzalez, S. F. Tapert, M. C. Riedel, L. M. Squeglia, L. W. Hyde, M. D. Rosenberg, E. A. Earl, K. D. Howlett, F. C. Baker, M. Soules, J. Diaz, O. R. de Leon, W. K. Thompson, M. C. Neale, M. Herting, E. R. Sowell, R. P. Alvarez, S. W. Hawes, M. Sanchez, J. Bodurka, F. J. Breslin, A. S. Morris, M. P. Paulus, W. K. Simmons, J. R. Polimeni, A. van der Kouwe, A. S. Nencka, K. M. Gray, C. Pierpaoli, J. A. Matochik, A. Noronha, W. M. Aklin, K. Conway, M. Glantz, E. Hoffman, R. Little, M. Lopez, V. Pariyadath, S. R. Weiss, D. L. Wolff-Hughes, R. Del Carmen-Wiggins, S. W. F. Ewing, O. Miranda-Dominguez, B. J. Nagel, A. J. Perrone, D. T. Sturgeon, A. Goldstone, A. Pfefferbaum, K. M. Pohl, D. Prouty, K. Uban, S. Y. Bookheimer, M. Dapretto, A. Galvan, K. Bagot, J. Giedd, M. A. Infante, J. Jacobus, K. Patrick, P. D. Shilling, R. Desikan, Y. Li, L. Sugrue, M. T. Banich, N. Friedman, J. K. Hewitt, C. Hopfer, J. Sakai, J. Tanabe, L. B. Cottler, S. J. Nixon, L. Chang, C. Cloak, T. Ernst, G. Reeves, D. N. Kennedy, S. Heeringa, S. Peltier, J. Schulenberg, C. Sripada, R. A. Zucker, W. G. Iacono, M. Luciana, F. J. Calabro, D. B. Clark, D. A. Lewis, B. Luna, C. Schirda, T. Brima, J. J. Foxe, E. G. Freedman, D. W. Mruzek, M. J. Mason, R. Huber, E. M. Glade, A. Prescot, P. F. Renshaw, D. A. Yurgelun-Todd, N. A. Allgaier, J. A. Dumas, M. Ivanova, A. Potter, P. Florsheim, C. Larson, K. Lisdahl, M. E. Charness, B. Fuemmeler, J. M. Hettema, H. H. Maes, J. Steinberg, A. P. Anokhin, P. Glaser, A. C. Heath, P. A. Madden, A. Baskin-Sommers, R. T. Constable, S. J. Grant, G. J. Dowling, S. A. Brown, T. L. Jernigan, A. M. Dale, Image processing and analysis methods for the Adolescent Brain Cognitive Development Study. *NeuroImage* **202**, 116091 (2019).
84. B. Fischl, FreeSurfer. *NeuroImage* **62**, 774–781 (2012).
85. B. Fischl, M. I. Sereno, R. B. H. Tootell, A. M. Dale, High-resolution intersubject averaging and a coordinate system for the cortical surface. *Hum. Brain Mapp.* **8**, 272–284 (1999).
86. D. N. Greve, B. Fischl, Accurate and robust brain image alignment using boundary-based registration. *Neuroimage* **48**, 63–72 (2009).
87. B. Fischl, M. I. Sereno, A. M. Dale, Cortical surface-based analysis. *NeuroImage* **9**, 195–207 (1999).
88. J. D. Power, A. Mitra, T. O. Laumann, A. Z. Snyder, B. L. Schlaggar, S. E. Petersen, Methods to detect, characterize, and remove motion artifact in resting state fMRI. *NeuroImage* **84**, 320–341 (2014).
89. B. Fischl, D. H. Salat, E. Busa, M. Albert, M. Dieterich, C. Haselgrove, A. van der Kouwe, R. Killiany, D. Kennedy, S. Klaveness, A. Montillo, N. Makris, B. Rosen, A. M. Dale, Whole brain segmentation: Automated labeling of neuroanatomical structures in the human brain. *Neuron* **33**, 341–355 (2002).
90. H. W. Kuhn, The Hungarian method for the assignment problem. *Nav. Res. Logist. Q.* **2**, 83–97 (1955).
91. J. Munkres, Algorithms for the assignment and transportation problems. *J. Soc. Ind. Appl. Math.* **5**, 32–38 (1957).
92. G. Varoquaux, P. R. Raamana, D. A. Engemann, A. Hoyos-Idrobo, Y. Schwartz, B. Thirion, Assessing and tuning brain decoders: Cross-validation, caveats, and guidelines. *NeuroImage* **145**, 166–179 (2017).
93. K. P. Murphy, *Machine Learning* (MIT Press, 2012).
94. A. M. Winkler, M. A. Webster, D. Vidaurre, T. E. Nichols, S. M. Smith, Multi-level block permutation. *NeuroImage* **123**, 253–268 (2015).
95. B. T. T. Yeo, F. M. Krienen, J. Sepulcre, M. R. Sabuncu, D. Lashkari, M. Hollinshead, J. L. Roffman, J. W. Smoller, L. Zöllei, J. R. Polimeni, B. Fischl, H. Liu, R. L. Buckner, The organization of the human cerebral cortex estimated by intrinsic functional connectivity. *J. Neurophysiol.* **106**, 1125–1165 (2011).

Acknowledgments: We would like to thank all the colleagues in INM-7 who have provided insightful comments and suggestions and all the reviewers for the thoughtful feedback. **Funding:** S.G. is supported by the Heisenberg Programme of the Deutsche Forschungsgemeinschaft (GE 2835/2–1); J.L., S.B.E., and K.R.P. are supported by the Deutsche Forschungsgemeinschaft (EI 816/4–1), the National Institute of Mental Health (R01-MH074457), the Helmholtz Portfolio Theme "Supercomputing and Modeling for the Human Brain," and the European Union's Horizon 2020 Research and Innovation Programme under grant agreement nos. 945539 (HBP SGA3) and 826421 (VirtualBrainCloud). B.T.T.Y., J.C., A.T., and L.Q.R.O. are supported by the Singapore National Research Foundation (NRF) Fellowship (Class of 2017), the NUS Yong Loo Lin School of Medicine (NUHSRO/2020/124/TMR/LOA), the Singapore National Medical Research Council (NMRC) LCG (OFLCG19May-0035), NMRC STaR (STaR20nov-0003), and the NIH (R01MH120080). Our computational work was partially performed on resources of the National Supercomputing Centre, Singapore (www.nscc.sg). Any opinions, findings, and conclusions or recommendations expressed in this material are those of the authors and do not reflect the views of the Singapore NRF or the Singapore NMRC. Data were provided by the Human Connectome Project, WU-Minn Consortium (principal investigators: D. V. Essen and K. Ugurbil; 1U54MH091657) funded by the 16 NIH Institutes and Centers that support the NIH Blueprint for Neuroscience Research, and by the McDonnell Center for Systems Neuroscience at Washington University. Data used in the preparation of this article were also obtained from the Adolescent Brain Cognitive Development[SM] (ABCD) Study (https://abcdstudy.org), held in the NIMH Data Archive (NDA). This is a multisite, longitudinal study designed to recruit more than 10,000 children of ages 9 to 10 and follow them over 10 years into early adulthood. The ABCD Study is supported by the NIH and additional federal partners under the following award numbers: U01DA041048, U01DA050989, U01DA051016, U01DA041022, U01DA051018, U01DA051037, U01DA050987, U01DA041174, U01DA041106, U01DA041117, U01DA041028, U01DA041134, U01DA050988, U01DA051039, U01DA041156, U01DA041025, U01DA041120, U01DA051038, U01DA041148, U01DA041093, U01DA041089, U24DA041123, and U24DA041147. A full list of supporters is available at https://abcdstudy.org/federal-partners.html. A listing of participating sites and a complete listing of the study investigators can be found at https://abcdstudy.org/consortium_members/. ABCD consortium investigators designed and implemented the study and/or provided data but did not necessarily participate in the analysis or writing of this report. This manuscript reflects the views of the authors and may not reflect the opinions or views of the NIH or ABCD consortium investigators. **Author contributions:** J.L., D.B., B.T.T.Y., and S.G. designed the research; J.L., J.C., A.T., and L.Q.R.O. collected and preprocessed the data; J.L. performed the data analyses; J.L. and S.G. drafted the article; D.B., A.J.H., T.G., K.R.P., M.J., S.B.E., and B.T.T.Y. provided critical review of the article; B.T.T.Y. contributed reagents and analytic tools. **Competing interests:** The authors declare that they have no competing interests. **Data and materials availability:** Both the HCP (www.humanconnectome.org/) and ABCD (http://dx.doi.org/10.15154/1504041) datasets are publicly available. Secondary data generated from this study can be found at https://doi.org/10.7910/DVN/SKXD1L and http://dx.doi.org/10.15154/1524287. The code specifically for the data analyses of this work was released on Dataverse: https://doi.org/10.7910/DVN/KPAMEE; https://doi.org/10.7910/DVN/WEJC0R. The current study also borrowed some code from our previous works, including the fMRI preprocessing pipeline of the ABCD dataset (https://github.com/ThomasYeoLab/CBIG/tree/master/stable_projects/preprocessing/CBIG_fMRI_Preproc2016), the Schaefer parcellation (https://github.com/ThomasYeoLab/CBIG/tree/master/stable_projects/brain_parcellation/Schaefer2018_LocalGlobal), the general kernel ridge regression package (https://github.com/ThomasYeoLab/CBIG/tree/master/utilities/matlab/predictive_models/KernelRidgeRegression), and the general linear ridge regression package (https://github.com/ThomasYeoLab/CBIG/tree/master/utilities/matlab/predictive_models/LRR_fitrlinear). A copy of the CBIG GitHub repository can also be found on Dataverse: https://doi.org/10.7910/DVN/TW9BBI. All data needed to evaluate the conclusions in the paper are present in the paper and/or the Supplementary Materials.

Submitted 27 April 2021
Accepted 26 January 2022
Published 16 March 2022
10.1126/sciadv.abj1812
