# Supplementary material for: Cross-ethnicity/race generalization failure of behavioral prediction from resting-state functional connectivity
Source: Sci Adv. 2022 Mar 16;8(11):eabj1812. doi: 10.1126/sciadv.abj1812 (PMC8926333; doi:10.1126/sciadv.abj1812)
Supplement: Supplementary file 2 — Supplementary Methods Tables S1 to S3 Figs. S1 to S11 [file sciadv.abj1812_sm.pdf]

CORRECTED 27 MAY 2022; SEE ERRATUM

Science Advances
AAAS

Supplementary Materials for

Cross-ethnicity/race generalization failure of behavioral prediction from resting-state functional connectivity

Jingwei Li*, Danilo Bzdok, Jianzhong Chen, Angela Tam, Leon Qi Rong Ooi, Avram J. Holmes, Tian Ge, Kaustubh R. Patil, Mbemba Jabbi, Simon B. Eickhoff, B. T. Thomas Yeo*, Sarah Genon*

*Corresponding author. Email: jingweili.sjtu.nus@gmail.com (J.L.); s.genon@fz-juelich.de (S.G.); thomas.yeo@nus.edu.sg (B.T.T.Y.)

Published 16 March 2022, *Sci. Adv.* **8**, eabj1812 (2022)
DOI: 10.1126/sciadv.abj1812

This PDF file includes:

Supplementary Methods
Tables S1 to S3
Figs. S1 to S11

Supplementary Materials

Supplementary Methods

In this section, we describe the mathematical details of kernel ridge regression. Let y_i^{train} denote the score of a behavioral measure of training participant i. Let c_i^{train} denote the vectorized RSFC (i.e., lower triangular entries of the RSFC matrix) of training participant i. The RSFC-based similarity between two training participants i and j can be denoted as $K\left(c_i^{train}, c_j^{train}\right) = corr\left(c_i^{train}, c_j^{train}\right)$, where $corr(\cdot)$ represents Pearson's correlation. Suppose there are N_1 training participants. Let K be an $N_1 \times N_1$ matrix, where the i-th row and j-th column of K is $K\left(c_i, c_j\right)$. Therefore, K is the similarity matrix among the N_1 training participants. In the training phase for a specific behavioral measure, the regression coefficient α is estimated from the training participants via Eq. S1:

$$\alpha = (K + \lambda I)^{-1} y^{train} \qquad (S1),$$

where y^{train} is the $N_1 \times 1$ vector containing the behavioral scores of all training participants. In this work, the regularization hyperparameter λ was selected by 10-fold cross-validation within the training set (i.e., inner-loop cross-validation).
Suppose the RSFC similarity between a test participant s and each training participant is consolidated into a $1 \times N_1$ vector $K_s = \left[corr\left(c_s^{test}, c_1^{train}\right), corr\left(c_s^{test}, c_2^{train}\right), \ldots, corr\left(c_s^{test}, c_{N_1}^{train}\right)\right]$, where c_s^{test} is the vectorized RSFC of the test participant. The predicted behavioral score of this test participant will be:

$$\hat{y}_s^{test} = K_s \alpha = K_s (K + \lambda I)^{-1} y^{train} \qquad (S2)$$

Supplementary Figures and Tables

**Description**	**HCP field**
Visual Episodic Memory	PicSeq_Unadj
Cognitive Flexibility (DCCS)	CardSort_Unadj
Inhibition (Flanker Task)	Flanker_Unadj
Fluid Intelligence (PMAT)	PMAT24_A_CR
Reading (Pronunciation)	ReadEng_Unadj
Vocabulary (Picture Matching)	PicVocab_Unadj
Processing Speed	ProcSpeed_Unadj
Delay Discounting	DDic_AUC_40K

Spatial Orientation	VSPLOT_TC
Sustained Attention – Sens.	SCPT_SEN
Sustained Attention – Spec.	SCPT_SPEC
Verbal Episodic Memory	IWRD_TOT
Working Memory (List Sorting)	ListSort_Unadj
Cognitive Status (MMSE)	MMSE_Score
Sleep Quality (PSQI)	PSQI_Score
Walking Endurance	Endurance_Unadj
Walking Speed	GaitSpeed_Unadj
Manual Dexterity	Dexterity_Unadj
Grip Strength	Strength_Unadj
Odor Identification	Odor_Unadj
Pain Interference Survey	PainInterf_Tscore
Taste Intensity	Taste_Unadj
Contrast Sensitivity	Mars_Final
Emotional Face Matching	Emotion_Task_Face_Acc
Arithmetic	Language_Task_Math_Avg_Difficulty_Level
Story Comprehension	Language_Task_Story_Avg_Difficulty_Level
Relational Processing	Relational_Task_Acc
Social Cognition – Random	Social_Task_Perc_Random
Social Cognition – Interaction	Social_Task_Perc_TOM
Working Memory (N-back)	WM_Task_Acc
Agreeableness (NEO)	NEOFAC_A
Openness (NEO)	NEOFAC_O
Conscientiousness (NEO)	NEOFAC_C
Neuroticism (NEO)	NEOFAC_N
Extraversion (NEO)	NEOFAC_E
Emot. Recog. – Total	ER40_CR
Emot. Recog. – Angry	ER40ANG
Emot. Recog. – Fear	ER40FEAR
Emot. Recog. – Happy	ER40HAP
Emot. Recog. - Neutral	ER40NOE
Emot. Recog. – Sad	ER40SAD
Anger – Affect	AngAffect_Unadj

Anger – Hostility	AngHostil_Unadj
Anger – Aggression	AngAggr_Unadj
Fear – Affect	FearAffect_Unadj
Fear – Somatic Arousal	FearSomat_Unadj
Sadness	Sadness_Unadj
Life Satisfaction	LifeSatisf_Unadj
Meaning & Purpose	MeanPurp_Unadj
Positive Affect	PosAffect_Unadj
Friendship	Friendship_Unadj
Loneliness	Loneliness_Unadj
Perceived Hostility	PercHostil_Unadj
Perceived Rejection	PercReject_Unadj
Emotional Support	EmotSupp_Unadj
Instrument Support	InstruSupp_Unadj
Perceived Stress	PercStress_Unadj
Self-Efficacy	SelfEff_Unadj

Table S1. Lookup table showing the original HCP variable names with the corresponding descriptive labels used in the manuscript.

Scale	Description	ABCD field
Rey Auditory Verbal Learning Test (RAVLT)	Short delay recall	pea_ravlt_sd_trial_vi_tc
	Long delay recall	pea_ravlt_ld_trial_vii_tc
Wechsler Intelligence Scale for Children-V (WISC-V)	Matrix reasoning	pea_wiscv_trs
NIH Toolbox	Cognitive control / Attention (Flanker)	nihtbx_flanker_uncorrected
	Working memory (list sort)	nihtbx_list_uncorrected
	Executive function (card sort)	nihtbx_cardsort_uncorrected
	Reading (pronunciation)	nihtbx_reading_uncorrected
	Processing speed	nihtbx_pattern_uncorrected
	Visual episodic memory	nihtbx_picture_uncorrected
	Picture vocabulary	nihtbx_picvocab_uncorrected
	Fluid cognition	nihtbx_fluidcomp_uncorrected
	Crystallized cognition	nihtbx_cryst_uncorrected
	Overall cognition	nihtbx_totalcomp_uncorrected

Little Man Task	Visuospatial accuracy	lmt_scr_perc_correct
	Visuospatial reaction time	lmt_scr_rt_correct
	Visuospatial efficiency	lmt_scr_efficiency
Achenbach Child Behavior Check List	Anxious/Depressed	cbcl_scr_syn_anxdep_r
	Withdrawn/Depressed	cbcl_scr_syn_withdep_r
	Somatic complaints	cbcl_scr_syn_somatic_r
	Social problems	cbcl_scr_syn_social_r
	Thought problems	cbcl_scr_syn_thought_r
	Attention problems	cbcl_scr_syn_attention_r
	Rule-breaking behavior	cbcl_scr_syn_rulebreak_r
	Aggressive behavior	cbcl_scr_syn_aggressive_r
Parent General Behavior Inventory	Mania	pgbi_p_ss_score
Pediatric Psychosis Questionnaire − Brief Version	Total prodromal psychosis symptoms	pps_y_ss_number
	Prodromal psychosis severity	pps_y_ss_severity_score
Modified UPPS-P for Children from PhenX	Negative urgency	upps_y_ss_negative_urgency
	Positive urgency	upps_y_ss_positive_urgency
	Lack of planning	upps_y_ss_lack_of_planning
	Lack of perseverance	upps_y_ss_lack_of_perseverance
	Sensation seeking	upps_y_ss_sensation_seeking
Behavioral Inhibition & Activation	Behavioral inhibition	bis_y_ss_bis_sum
	BAS - Reward responsiveness	bis_y_ss_bas_rr
	BAS - Drive	bis_y_ss_bas_drive
	BAS - Fun seeking	bis_y_ss_bas_fs

Table S2. Summary of ABCD behavioral measures with the descriptive names used in the manuscript.

HCP behavioral name	Mode of regularization hyperparameter		ABCD behavioral name	Mode of regularization hyperparameter	
	Kernel	Linear		Kernel	Linear
Visual Episodic Memory	3	1.2376x10^3	Short delay recall	10	1.2376x10^3
Cognitive Flexibility (DCCS)	2	1.2376x10^3	Long delay recall	5	1.2376x10^3
Inhibition (Flanker task)	4	1.2376x10^3	Matrix reasoning	5	1.2376x10^3
Reading (Pronunciation)	0.7	1.2376x10^3	Cognitive control / Attention (Flanker)	10	1.2376x10^3

Processing Speed	4	$1.2376x10^3$	Working memory (list sort)	4	$1.2376x10^3$
Spatial Orientation	1	$1.2376x10^3$	Executive function (card sort)	10	$1.2376x10^3$
Sustained Attention - Sens.	20	$5.2750x10^7$	Reading (pronunciation)	2.5	$1.2376x10^3$
Sustained Attention - Spec.	10	$1.2376x10^3$	Processing speed	10	$1.2376x10^3$
Verbal Episodic Memory	10	$1.2376x10^3$	Visual episodic memory	5	$1.2376x10^3$
Working Memory (List Sorting)	3	$1.2376x10^3$	Picture vocabulary	2.5	$1.2376x10^3$
Cognitive Status (MMSE)	20	$5.2750x10^7$	Fluid cognition	3	$1.2376x10^3$
Sleep Quality (PSQI)	5	$1.2376x10^3$	Crystallized cognition	2	$1.2376x10^3$
Walking Endurance	5	$1.2376x10^3$	Overall cognition	2	$1.2376x10^3$
Walking Speed	10	$1.2376x10^3$	Visuospatial accuracy	5	$1.2376x10^3$
Manual Dexterity	20	$5.2750x10^7$	Visuospatial reaction time	20	$1.2376x10^3$
Grip Strength	1.5	$1.2376x10^3$	Visuospatial efficiency	10	$1.2376x10^3$
Odor Identification	20	$5.2750x10^7$	Anxious/Depressed	20	$1.2376x10^3$
Pain Interference Survey	20	$5.2750x10^3$	Withdrawn/Depressed	20	$1.2376x10^3$
Taste Intensity	20	$1.2376x10^3$	Somatic complaints	20	$1.2376x10^3$
Contrast Sensitivity	10	$1.2376x10^3$	Social problems	20	$1.2376x10^3$
Emotional Face Matching	20	$1.2376x10^3$	Thought problems	20	$1.2376x10^3$
Arithmetic	5	$1.2376x10^3$	Attention problems	10	$1.2376x10^3$
Social Cognition – Random	20	$1.2376x10^3$	Rule-breaking behavior	15	$1.2376x10^3$
Social Cognition – Interaction	20	$1.2376x10^3$	Aggressive behavior	20	$1.2376x10^3$
Agreeableness (NEO)	20	$1.2376x10^3$	Mania	20	$1.2376x10^3$
Openness (NEO)	1.5	$1.2376x10^3$	Total prodromal psychosis symptoms	15	$1.2376x10^3$
Conscientiousness (NEO)	5	$1.2376x10^3$	Prodromal psychosis severity	15	$1.2376x10^3$
Neuroticism (NEO)	10	$1.2376x10^3$	Negative urgency	20	$1.2376x10^3$
Extraversion (NEO)	2	$1.2376x10^3$	Positive urgency	20	$1.2376x10^3$
Emot. Recog. – Total	5	$5.2750x10^7$	Lack of planning	20	$1.2376x10^3$
Emot. Recog. – Angry	20	$1.2376x10^3$	Lack of perseverance	20	$1.2376x10^3$
Emot. Recog. – Fear	20	$5.2750x10^7$	Sensation seeking	20	$1.2376x10^3$

Emot. Recog. – Happy	20	1.2376x10^3	Behavioral inhibition	20	1.2376x10^3
Emot. Recog. – Neutral	20	5.2750x10^7	BAS - Reward responsiveness	20	1.2376x10^3
Emot. Recog. – Sad	20	5.2750x10^3	BAS - Drive	20	1.2376x10^3
Anger – Affect	20	1.2376x10^3	BAS - Fun seeking	20	1.2376x10^3
Anger – Hostility	20	1.2376x10^3			
Anger – Aggression	10	1.2376x10^3			
Fear – Affect	10	1.2376x10^3			
Fear - Somatic Arousal	20	1.2376x10^3			
Sadness	5	1.2376x10^3			
Meaning & Purpose	5	1.2376x10^3			
Positive Affect	20	1.2376x10^3			
Friendship	5	1.2376x10^3			
Loneliness	3	1.2376x10^3			
Perceived Hostility	20	5.2750x10^7			
Perceived Rejection	20	1.2376x10^3			
Emotional Support	20	1.2376x10^3			
Instrument Support	5	5.2750x10^7			
Perceived Stress	5	1.2376x10^3			
Self-Efficacy	5	1.2376x10^3			

Table S3. Optimal hyperparameter for each behavioral measure using kernel ridge regression and linear ridge regression. Mode values were reported here across 40 random data splits and 10 cross-validation folds for the HCP dataset, and across 120 training-test splits for the ABCD dataset.

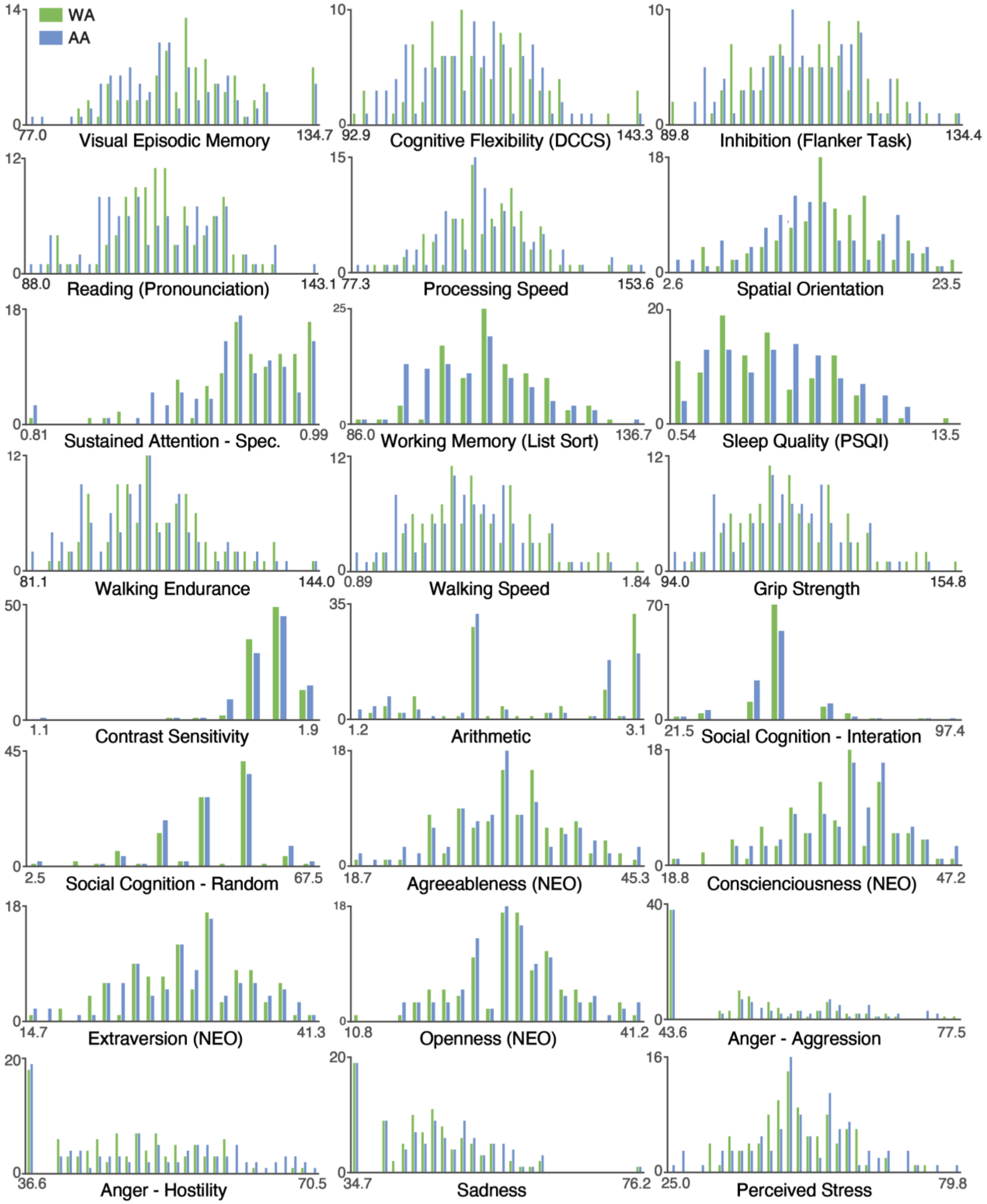

Figure S1. Distributions of exemplar HCP behavioral measures in matched AA and WA. For each behavioral measure, a representative data split of the total 40 random data splits is illustrated. Green: WA; blue: AA.

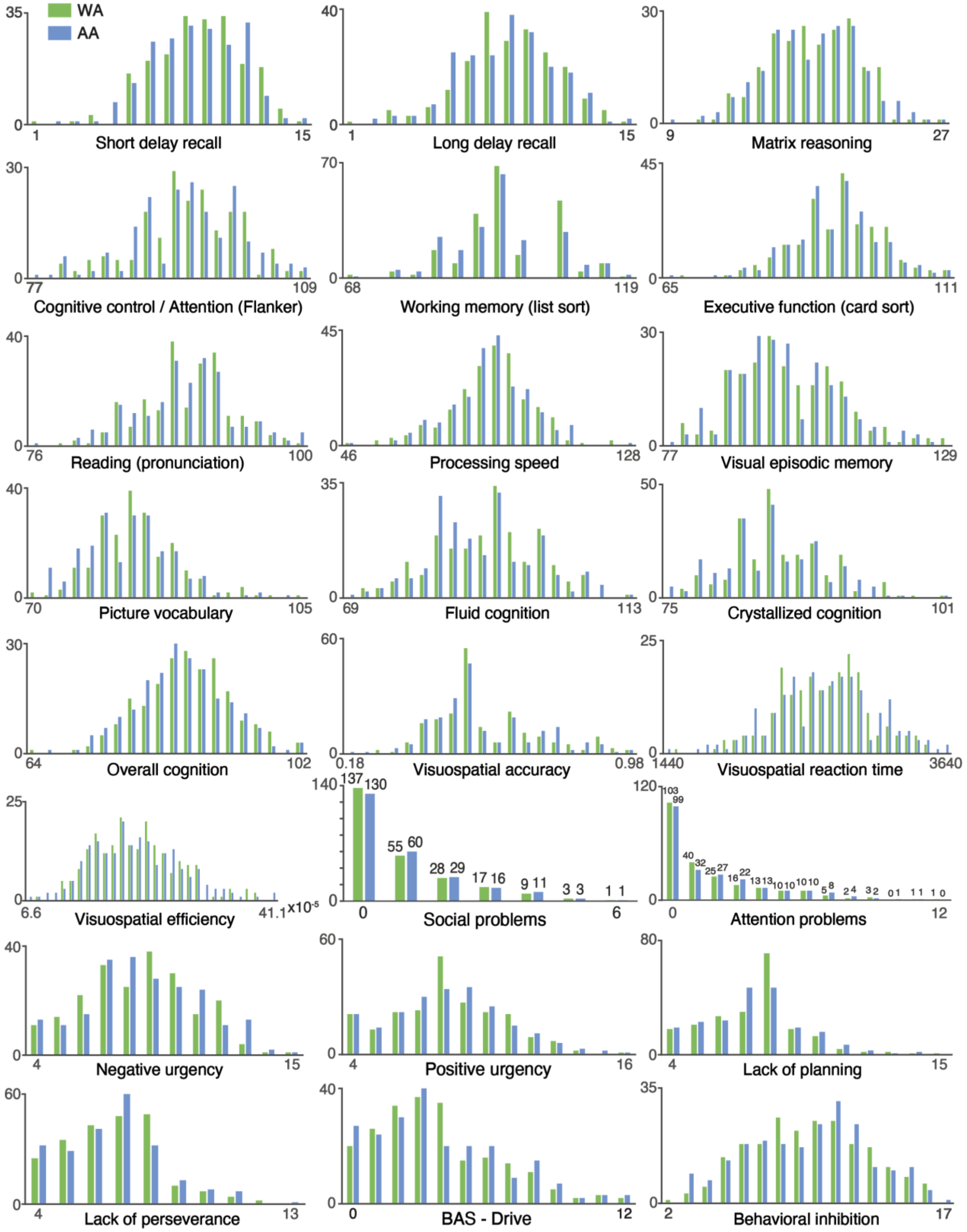

Figure S2. Distributions of exemplar ABCD behavioral measures in matched AA and WA. Green: WA; blue: AA.

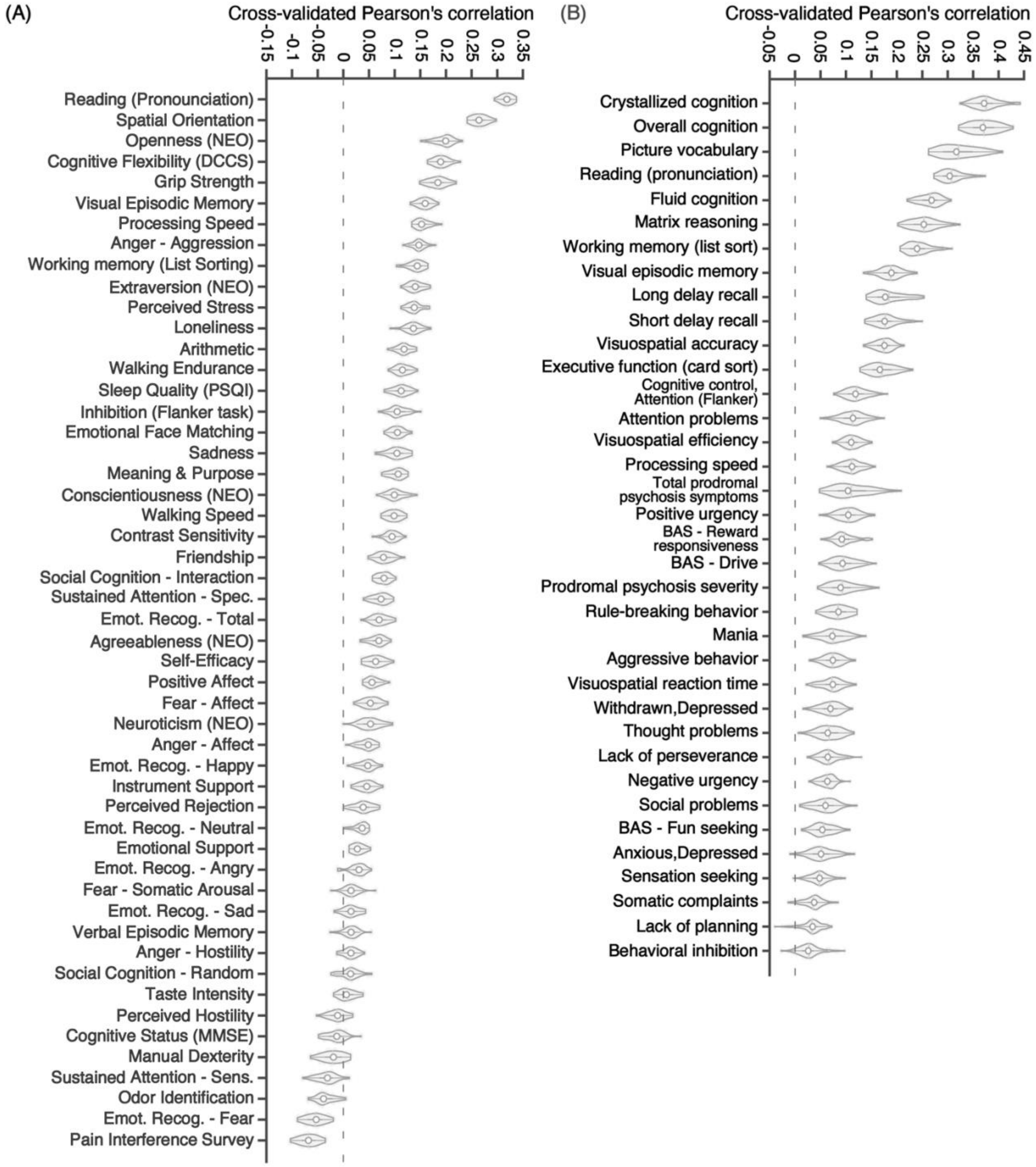

Figure S3. Pearson's correlation accuracy across all test participants from every ethnic/racial group. (A) HCP dataset. Each violin plot shows the variation across 40 data splits. (B) ABCD dataset. Each violin plot shows the variation across 120 training-test splits.

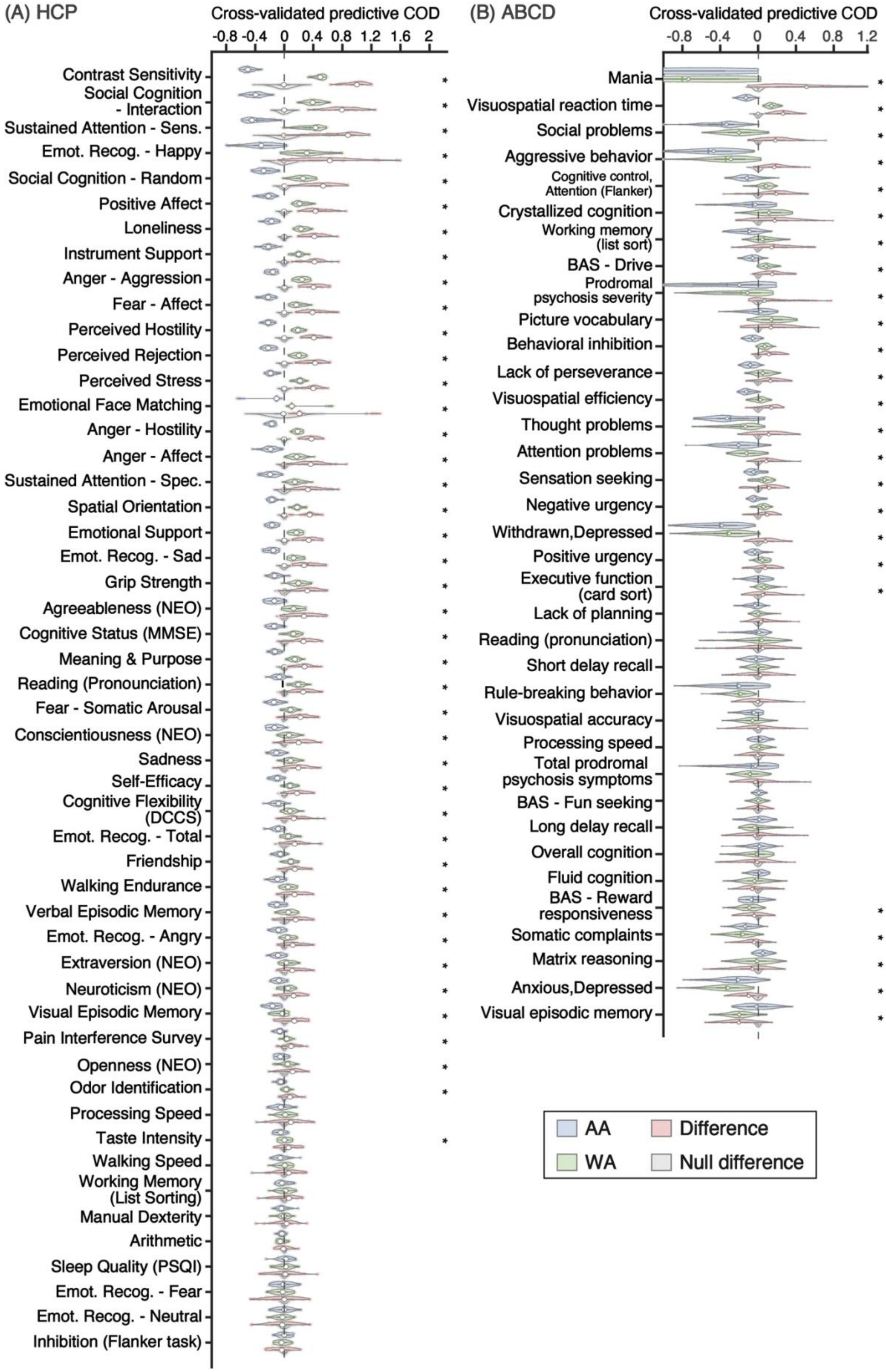
(A) HCP
Cross-validated predictive COD
-0.8 -0.4 0 0.4 0.8 1.2 1.6 2
Contrast Sensitivity
Social Cognition - Interaction
Sustained Attention - Sens.
Emot. Recog. - Happy
Social Cognition - Random
Positive Affect
Loneliness
Instrument Support
Anger - Aggression
Fear - Affect
Perceived Hostility
Perceived Rejection
Perceived Stress
Emotional Face Matching
Anger - Hostility
Anger - Affect
Sustained Attention - Spec.
Spatial Orientation
Emotional Support
Emot. Recog. - Sad
Grip Strength
Agreeableness (NEO)
Cognitive Status (MMSE)
Meaning & Purpose
Reading (Pronounciation)
Fear - Somatic Arousal
Conscientiousness (NEO)
Sadness
Self-Efficacy
Cognitive Flexibility (DCCS)
Emot. Recog. - Total
Friendship
Walking Endurance
Verbal Episodic Memory
Emot. Recog. - Angry
Extraversion (NEO)
Neuroticism (NEO)
Visual Episodic Memory
Pain Interference Survey
Openness (NEO)
Odor Identification
Processing Speed
Taste Intensity
Walking Speed
Working Memory (List Sorting)
Manual Dexterity
Arithmetic
Sleep Quality (PSQI)
Emot. Recog. - Fear
Emot. Recog. - Neutral
Inhibition (Flanker task)
(B) ABCD
Cross-validated predictive COD
-0.8 -0.4 0 0.4 0.8 1.2
Mania
Visuospatial reaction time
Social problems
Aggressive behavior
Cognitive control, Attention (Flanker)
Crystallized cognition
Working memory (list sort)
BAS - Drive
Prodromal psychosis severity
Picture vocabulary
Behavioral inhibition
Lack of perseverance
Visuospatial efficiency
Thought problems
Attention problems
Sensation seeking
Negative urgency
Withdrawn,Depressed
Positive urgency
Executive function (card sort)
Lack of planning
Reading (pronunciation)
Short delay recall
Rule-breaking behavior
Visuospatial accuracy
Processing speed
Total prodromal psychosis symptoms
BAS - Fun seeking
Long delay recall
Overall cognition
Fluid cognition
BAS - Reward responsiveness
Somatic complaints
Matrix reasoning
Anxious,Depressed
Visual episodic memory
AA
Difference
WA
Null difference

Figure S4. Matched AA suffered from larger prediction error (i.e., lower predictive COD) than matched WA using kernel ridge regression (showing all behavioral measures). (A) HCP dataset. Each violin plot shows the various predictive COD across 40 data splits. (B) ABCD dataset. Each violin plot shows the various predictive COD across 120 training-test splits. Blue and green violins represent AA and WA, respectively. Red violins are the difference. Grey violins show the null distribution of difference generated by randomly flipping the AA vs. WA labels. * indicates that the difference in predictive COD between matched AA and WA was significant (FDR controlled at 5%). Grey dashed line indicates 0.

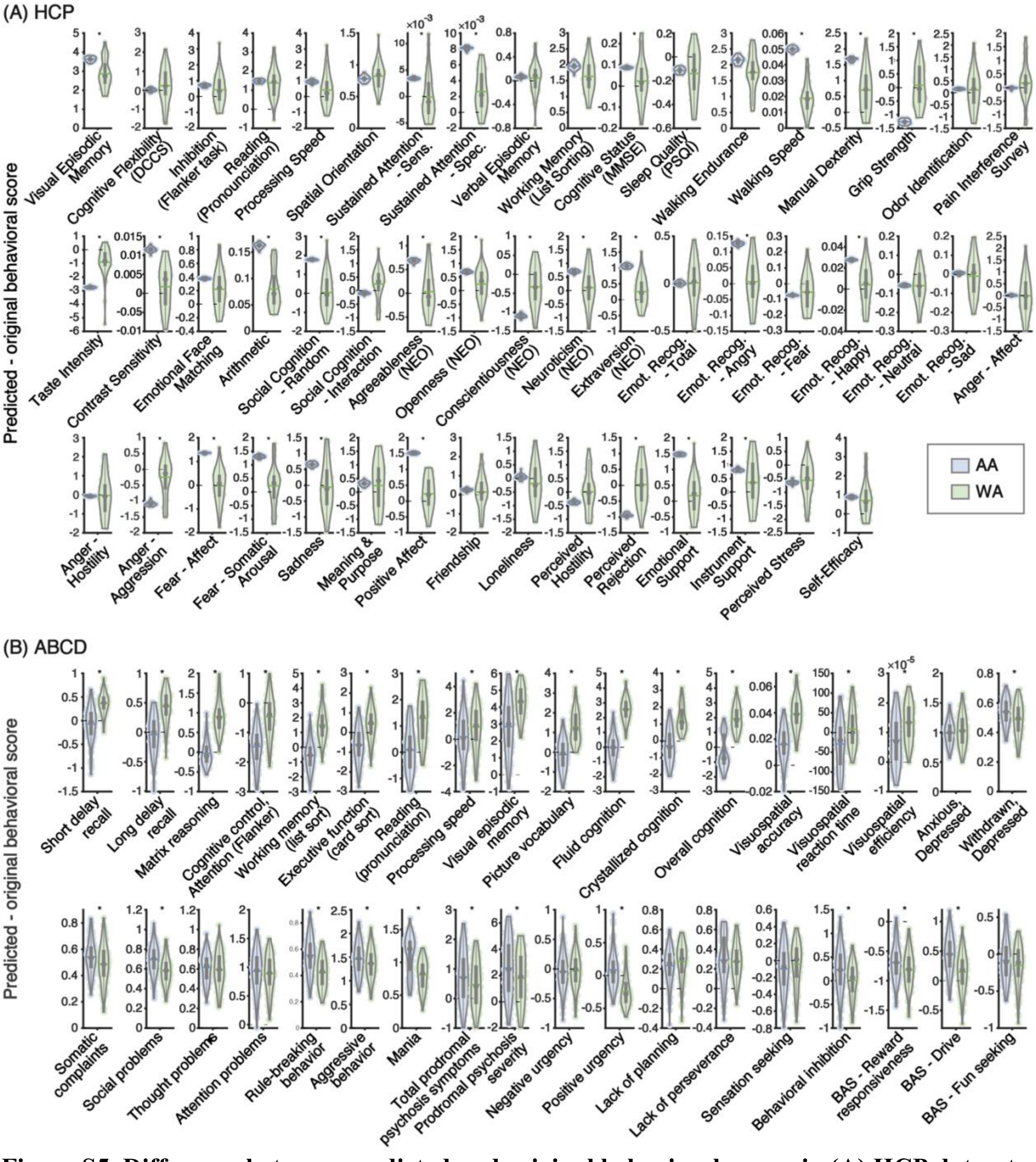

Figure S5. Difference between predicted and original behavioral scores in (A) HCP dataset and (B) ABCD dataset. Each violin plot shows the variation across 40 random splits in (A) and

the variation across 120 training-test splits in (B). Blue and green violins correspond to AA and WA respectively. A star above a subplot indicates that the mean values between AA and WA were significantly different. Note that the scale of vertical axes depends on the scale of the psychometric score resulting in different scales for different behavioral measures.

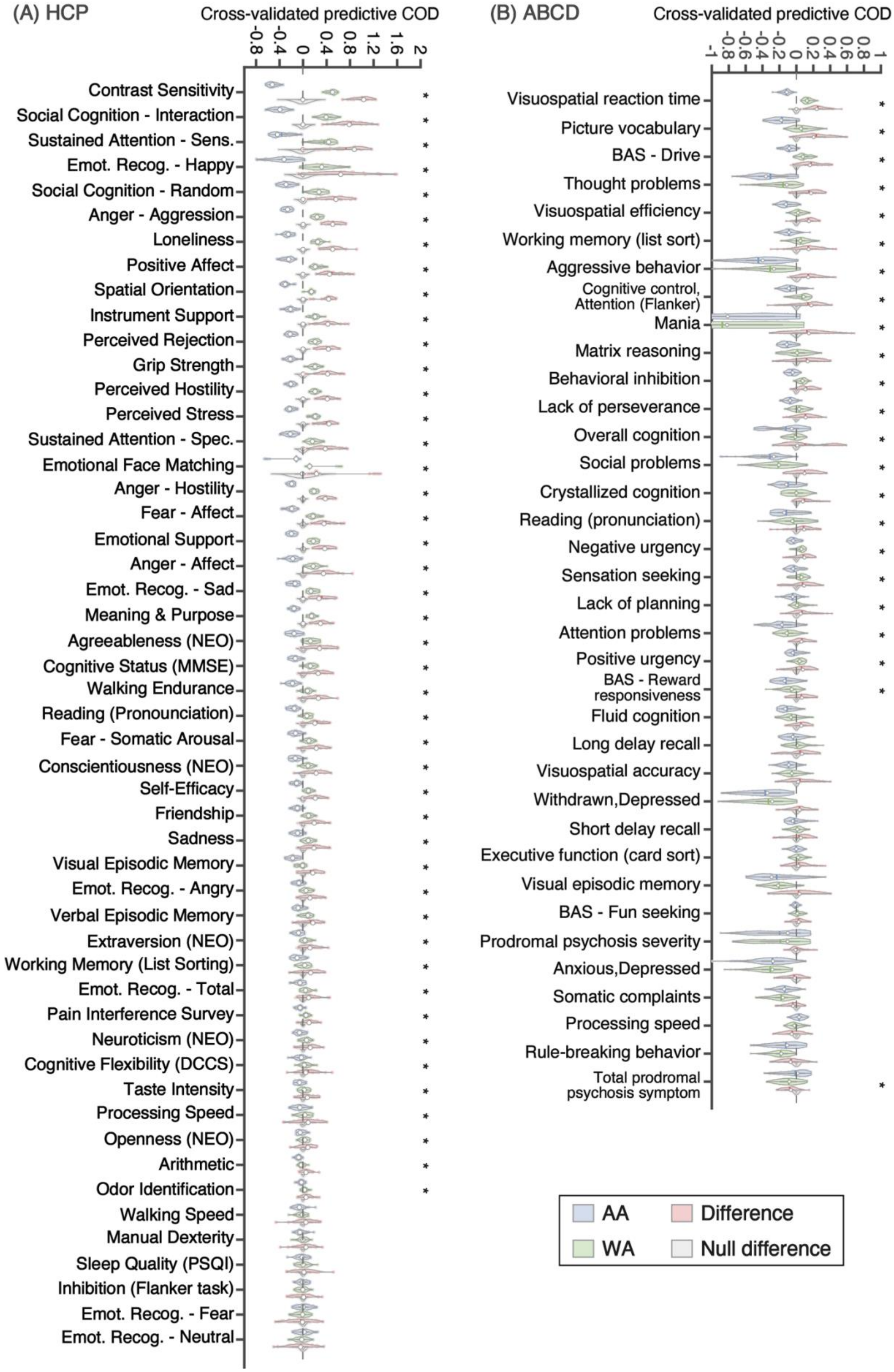

(A) HCP
Cross-validated predictive COD
-0.8
-0.4
0
0.4
0.8
1.2
1.6
2
Contrast Sensitivity
Social Cognition - Interaction
Sustained Attention - Sens.
Emot. Recog. - Happy
Social Cognition - Random
Anger - Aggression
Loneliness
Positive Affect
Spatial Orientation
Instrument Support
Perceived Rejection
Grip Strength
Perceived Hostility
Perceived Stress
Sustained Attention - Spec.
Emotional Face Matching
Anger - Hostility
Fear - Affect
Emotional Support
Anger - Affect
Emot. Recog. - Sad
Meaning & Purpose
Agreeableness (NEO)
Cognitive Status (MMSE)
Walking Endurance
Reading (Pronounciation)
Fear - Somatic Arousal
Conscientiousness (NEO)
Self-Efficacy
Friendship
Sadness
Visual Episodic Memory
Emot. Recog. - Angry
Verbal Episodic Memory
Extraversion (NEO)
Working Memory (List Sorting)
Emot. Recog. - Total
Pain Interference Survey
Neuroticism (NEO)
Cognitive Flexibility (DCCS)
Taste Intensity
Processing Speed
Openness (NEO)
Arithmetic
Odor Identification
Walking Speed
Manual Dexterity
Sleep Quality (PSQI)
Inhibition (Flanker task)
Emot. Recog. - Fear
Emot. Recog. - Neutral
(B) ABCD
Cross-validated predictive COD
-1
-0.8
-0.6
-0.4
-0.2
0
0.2
0.4
0.6
0.8
1
Visuospatial reaction time
Picture vocabulary
BAS - Drive
Thought problems
Visuospatial efficiency
Working memory (list sort)
Aggressive behavior
Cognitive control, Attention (Flanker)
Mania
Matrix reasoning
Behavioral inhibition
Lack of perseverance
Overall cognition
Social problems
Crystallized cognition
Reading (pronunciation)
Negative urgency
Sensation seeking
Lack of planning
Attention problems
Positive urgency
BAS - Reward responsiveness
Fluid cognition
Long delay recall
Visuospatial accuracy
Withdrawn,Depressed
Short delay recall
Executive function (card sort)
Visual episodic memory
BAS - Fun seeking
Prodromal psychosis severity
Anxious,Depressed
Somatic complaints
Processing speed
Rule-breaking behavior
Total prodromal psychosis symptom
AA
WA
Difference
Null difference

Figure S6. Matched AA suffered from larger prediction error (i.e., lower predictive COD) than matched WA using linear ridge regression (showing all behavioral measures). (A) HCP dataset. Each violin plot shows the various predictive COD across 40 data splits. (B) ABCD dataset. Each violin plot shows the various predictive COD across 120 training-test splits. Blue and green violins represent AA and WA, respectively. Red violins are the difference. Grey violins show the null distribution of difference generated by randomly flipping the AA vs. WA labels. * indicates that the difference in predictive COD between matched AA and WA was significant (FDR controlled at 5%). Grey dashed line indicates 0.

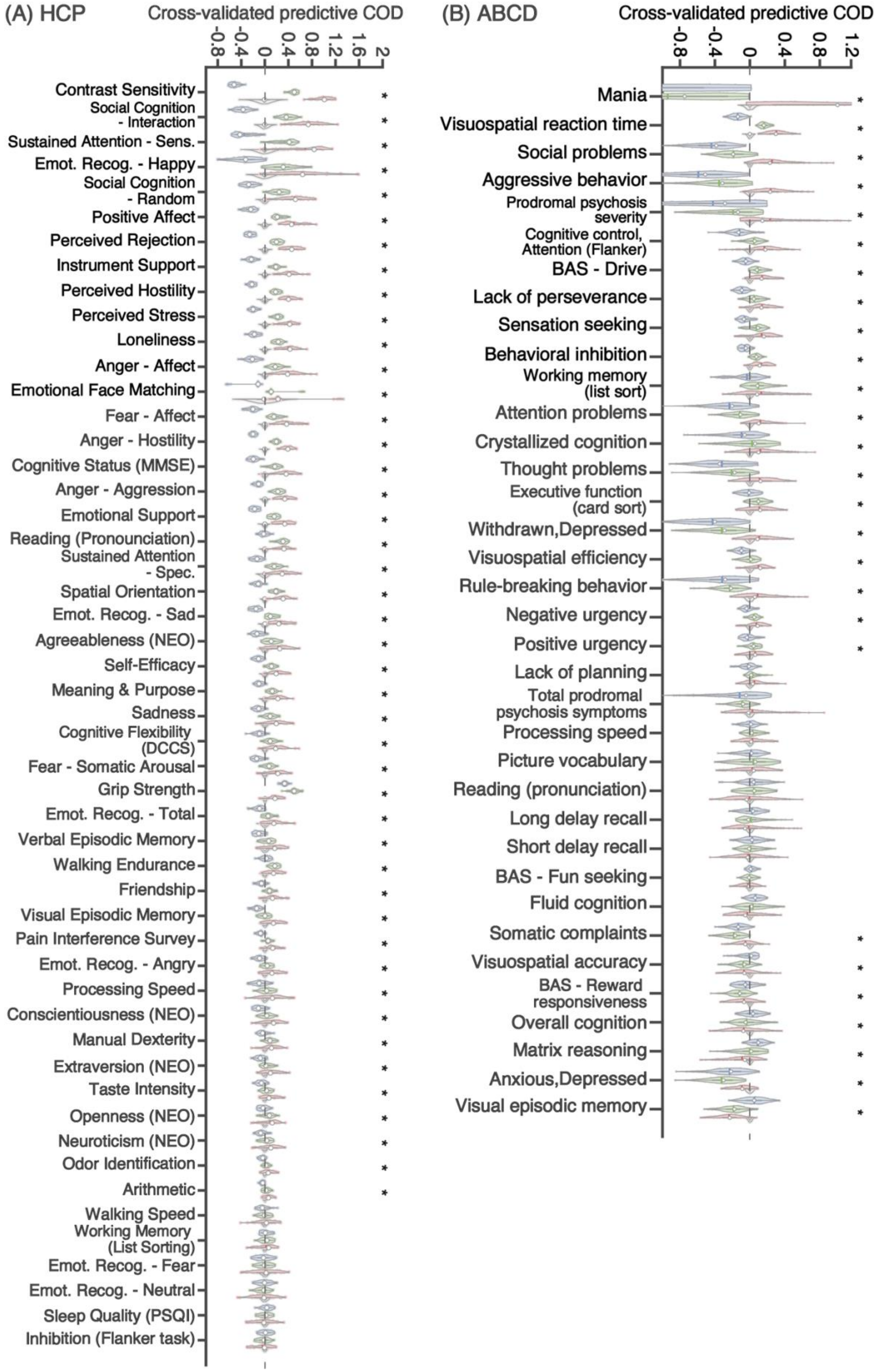
(A) HCP
Cross-validated predictive COD
-0.8
-0.4
0
0.4
0.8
1.2
1.6
2
Contrast Sensitivity
Social Cognition - Interaction
Sustained Attention - Sens.
Emot. Recog. - Happy
Social Cognition - Random
Positive Affect
Perceived Rejection
Instrument Support
Perceived Hostility
Perceived Stress
Loneliness
Anger - Affect
Emotional Face Matching
Fear - Affect
Anger - Hostility
Cognitive Status (MMSE)
Anger - Aggression
Emotional Support
Reading (Pronounciation)
Sustained Attention - Spec.
Spatial Orientation
Emot. Recog. - Sad
Agreeableness (NEO)
Self-Efficacy
Meaning & Purpose
Sadness
Cognitive Flexibility (DCCS)
Fear - Somatic Arousal
Grip Strength
Emot. Recog. - Total
Verbal Episodic Memory
Walking Endurance
Friendship
Visual Episodic Memory
Pain Interference Survey
Emot. Recog. - Angry
Processing Speed
Conscientiousness (NEO)
Manual Dexterity
Extraversion (NEO)
Taste Intensity
Openness (NEO)
Neuroticism (NEO)
Odor Identification
Arithmetic
Walking Speed
Working Memory (List Sorting)
Emot. Recog. - Fear
Emot. Recog. - Neutral
Sleep Quality (PSQI)
Inhibition (Flanker task)
(B) ABCD
Cross-validated predictive COD
-0.8
-0.4
0
0.4
0.8
1.2
Mania
Visuospatial reaction time
Social problems
Aggressive behavior
Prodromal psychosis severity
Cognitive control, Attention (Flanker)
BAS - Drive
Lack of perseverance
Sensation seeking
Behavioral inhibition
Working memory (list sort)
Attention problems
Crystallized cognition
Thought problems
Executive function (card sort)
Withdrawn,Depressed
Visuospatial efficiency
Rule-breaking behavior
Negative urgency
Positive urgency
Lack of planning
Total prodromal psychosis symptoms
Processing speed
Picture vocabulary
Reading (pronunciation)
Long delay recall
Short delay recall
BAS - Fun seeking
Fluid cognition
Somatic complaints
Visuospatial accuracy
BAS - Reward responsiveness
Overall cognition
Matrix reasoning
Anxious,Depressed
Visual episodic memory

Figure S7. Matched AA suffered from larger prediction error (i.e., lower predictive COD) than matched WA when no confounding variable was regressed during model building (showing all behavioral measures). (A) HCP dataset. Each violin plot shows the various predictive COD across 40 data splits. (B) ABCD dataset. Each violin plot shows the various predictive COD across 120 training-test splits. Blue and green violins represent AA and WA, respectively. Red violins are the difference. Grey violins show the null distribution of difference generated by randomly flipping the AA vs. WA labels. * indicates that the difference in predictive COD between matched AA and WA was significant (FDR controlled at 5%). Grey dashed line indicates 0.

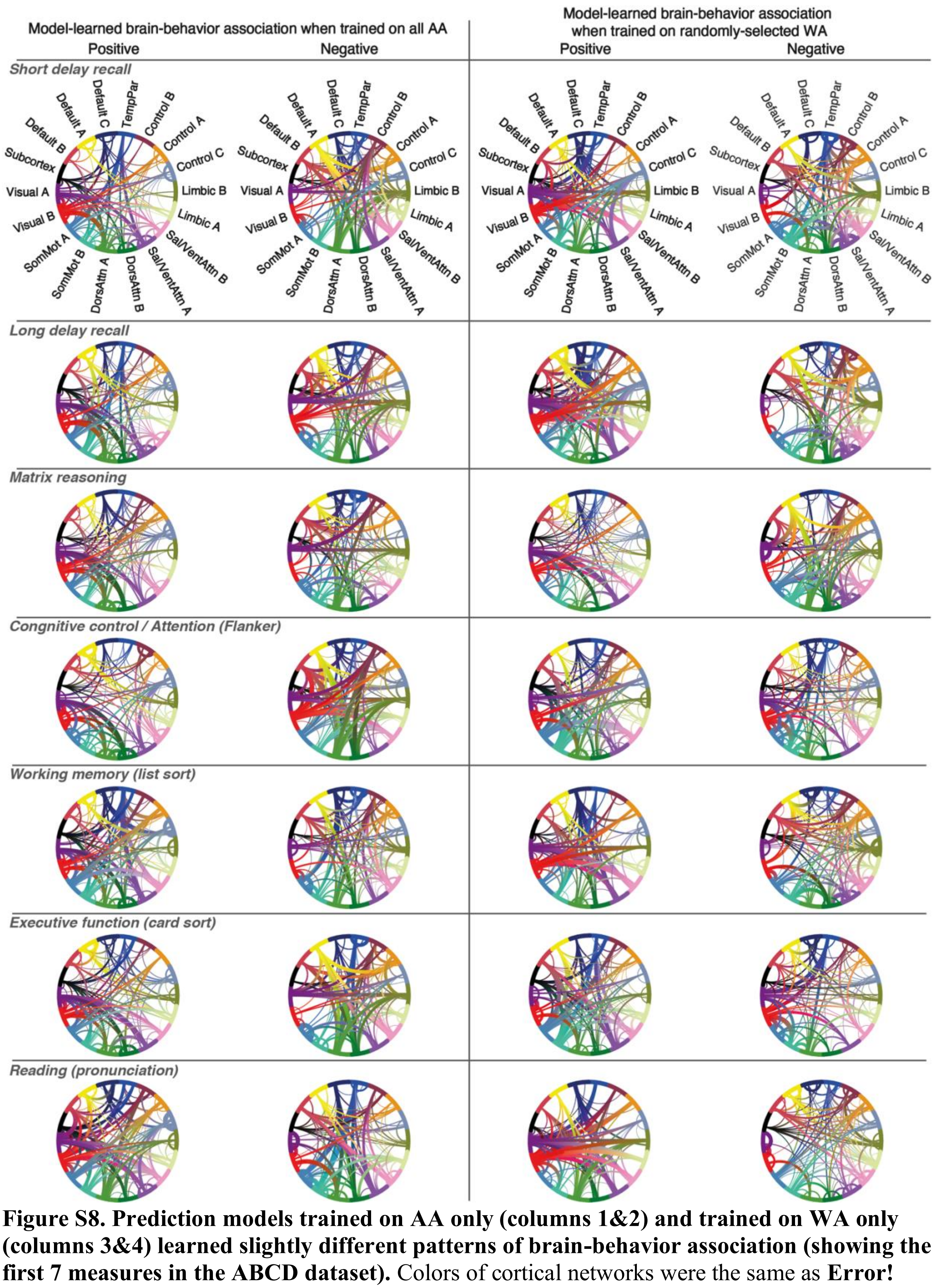

Figure S8. Prediction models trained on AA only (columns 1&2) and trained on WA only (columns 3&4) learned slightly different patterns of brain-behavior association (showing the first 7 measures in the ABCD dataset). Colors of cortical networks were the same as **Error! Reference source not found.**C.

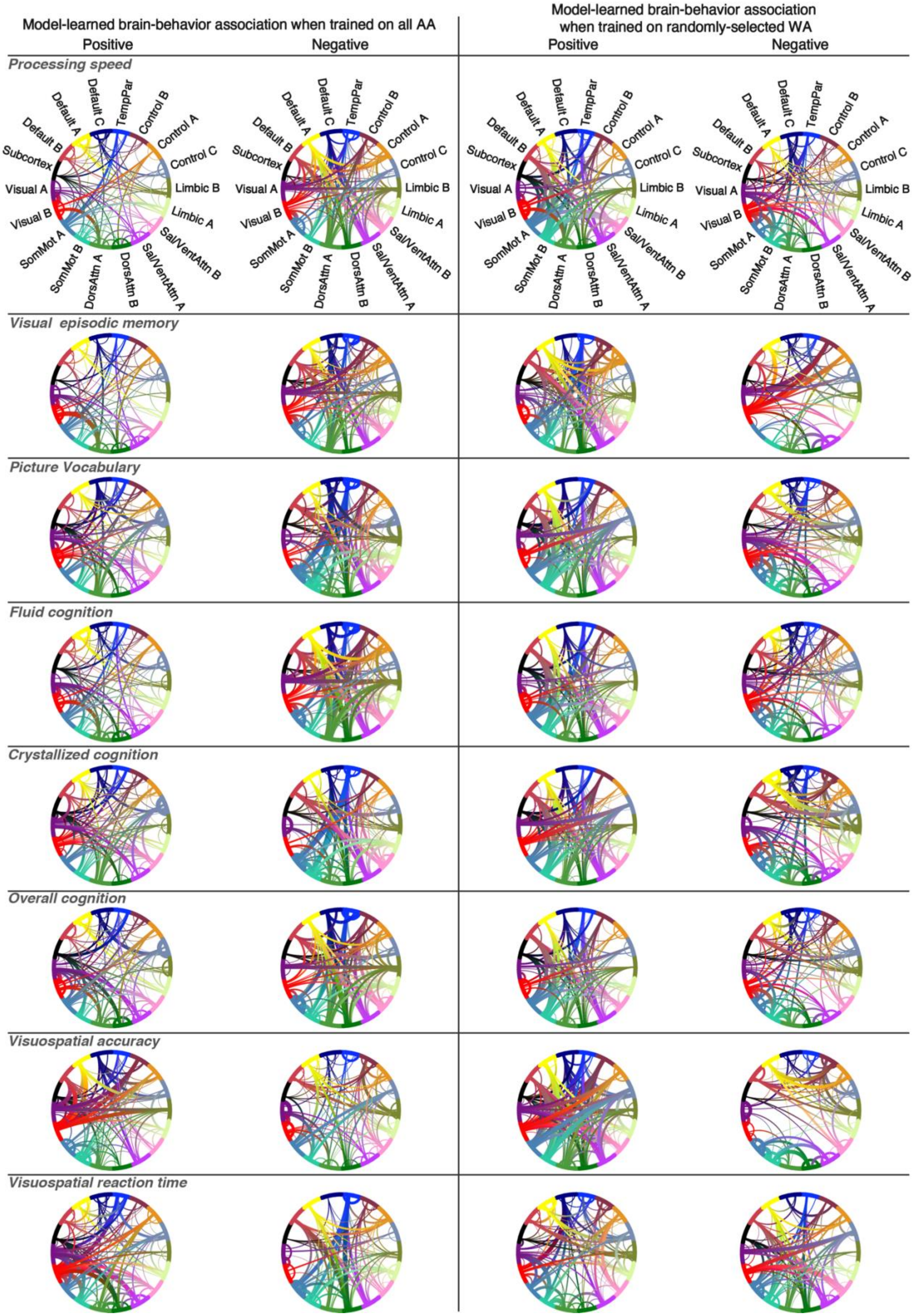

Model-learned brain-behavior association when trained on all AA
Model-learned brain-behavior association when trained on randomly-selected WA
Positive
Negative
Positive
Negative
Processing speed
Default C
TempPar
Control B
Default A
Control A
Default B
Control C
Subcortex
Visual A
Limbic B
Visual B
Limbic A
SomMot A
Sal/VentAttn B
SomMot B
DorsAttn A
DorsAttn B
Sal/VentAttn A
Visual episodic memory
Picture Vocabulary
Fluid cognition
Crystallized cognition
Overall cognition
Visuospatial accuracy
Visuospatial reaction time

Figure S9. Prediction models trained on AA only (columns 1&2) and trained on WA only (columns 3&4) learned slightly different patterns of brain-behavior association (continuing). Colors of cortical networks were the same as **Error! Reference source not found.**C.

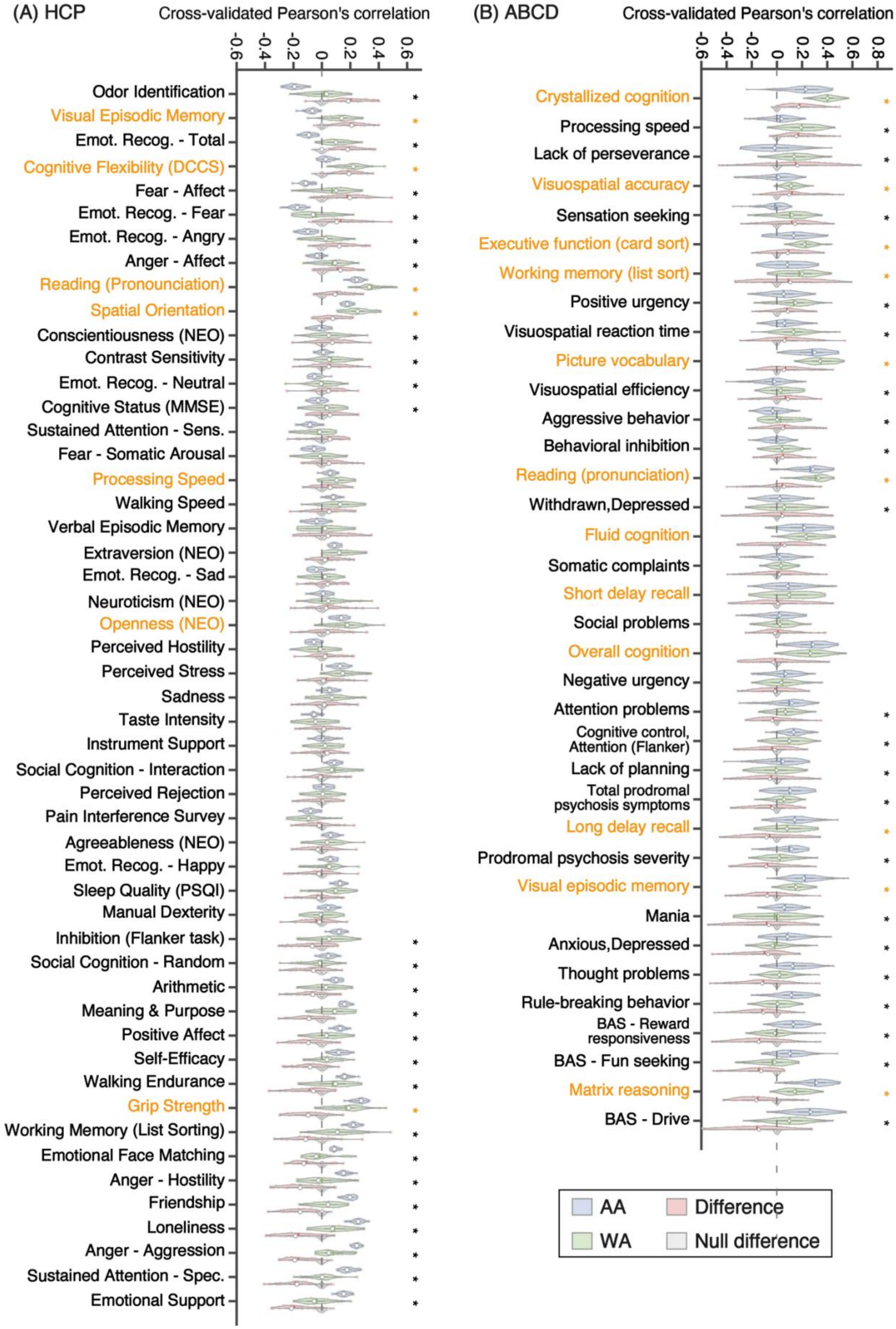
(A) HCP
Cross-validated Pearson's correlation
-0.6
-0.4
-0.2
0
0.2
0.4
0.6
Odor Identification
Visual Episodic Memory
Emot. Recog. - Total
Cognitive Flexibility (DCCS)
Fear - Affect
Emot. Recog. - Fear
Emot. Recog. - Angry
Anger - Affect
Reading (Pronounciation)
Spatial Orientation
Conscientiousness (NEO)
Contrast Sensitivity
Emot. Recog. - Neutral
Cognitive Status (MMSE)
Sustained Attention - Sens.
Fear - Somatic Arousal
Processing Speed
Walking Speed
Verbal Episodic Memory
Extraversion (NEO)
Emot. Recog. - Sad
Neuroticism (NEO)
Openness (NEO)
Perceived Hostility
Perceived Stress
Sadness
Taste Intensity
Instrument Support
Social Cognition - Interaction
Perceived Rejection
Pain Interference Survey
Agreeableness (NEO)
Emot. Recog. - Happy
Sleep Quality (PSQI)
Manual Dexterity
Inhibition (Flanker task)
Social Cognition - Random
Arithmetic
Meaning & Purpose
Positive Affect
Self-Efficacy
Walking Endurance
Grip Strength
Working Memory (List Sorting)
Emotional Face Matching
Anger - Hostility
Friendship
Loneliness
Anger - Aggression
Sustained Attention - Spec.
Emotional Support
(B) ABCD
Cross-validated Pearson's correlation
-0.6
-0.4
-0.2
0
0.2
0.4
0.6
0.8
Crystallized cognition
Processing speed
Lack of perseverance
Visuospatial accuracy
Sensation seeking
Executive function (card sort)
Working memory (list sort)
Positive urgency
Visuospatial reaction time
Picture vocabulary
Visuospatial efficiency
Aggressive behavior
Behavioral inhibition
Reading (pronunciation)
Withdrawn,Depressed
Fluid cognition
Somatic complaints
Short delay recall
Social problems
Overall cognition
Negative urgency
Attention problems
Cognitive control, Attention (Flanker)
Lack of planning
Total prodromal psychosis symptoms
Long delay recall
Prodromal psychosis severity
Visual episodic memory
Mania
Anxious,Depressed
Thought problems
Rule-breaking behavior
BAS - Reward responsiveness
BAS - Fun seeking
Matrix reasoning
BAS - Drive
AA
Difference
WA
Null difference

Figure S10. Differences in Pearson's correlation as the accuracy metric of full-dataset models between matched AA and WA in (A) the HCP dataset and (B) the ABCD dataset. For both datasets, confounds were regressed out from both RSFC and behavioral measures. Each violin plot shows the various correlation accuracy across 40 data splits in (A) and across 120 training-test splits in (B). The behavioral names in yellow indicate that they were significantly predictable and achieved a mean Pearson's correlation >0.15 between the predicted and true behavioral scores across all test participants including every ethnic/racial group. Blue and green violins represent AA and WA respectively. Red violin is the difference. Grey violin represents the null distribution of the difference generated by randomly flipping AA and WA labels. * indicates that the difference in correlation accuracy between matched AA and WA was significant (FDR controlled at 5%). Grey dashed line indicates 0.

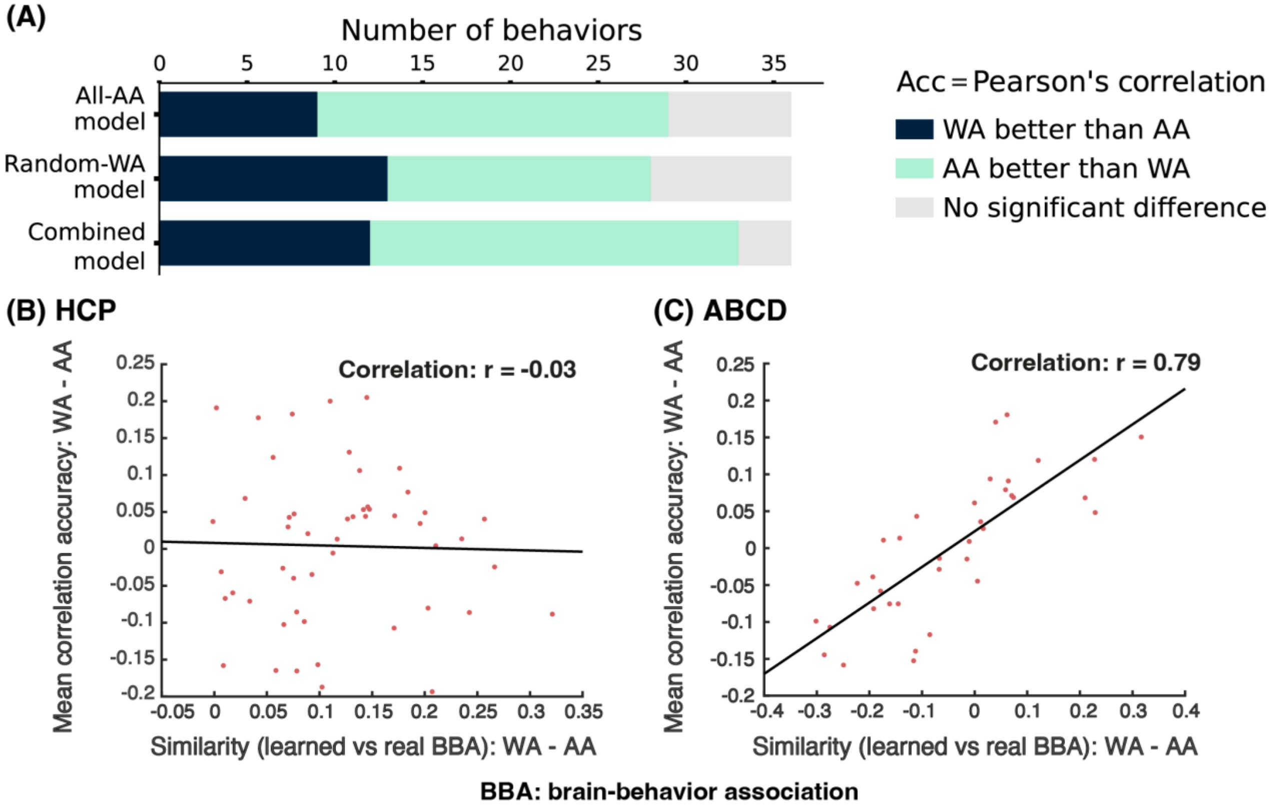

Figure S11. Extended analyses. (A) Impact of training population for behavioral prediction model. The influence of training population was evaluated using Pearson's correlation. Each bar corresponds to one of three types of prediction models: (1) trained on AA only; (2) trained on same number of random WA; (3) trained on both. For each model, the number of behavioral measures with better performance in WA than AA is indicated by the length of navy-blue bar, while mint color represents the number of behavioral variables with better performance in AA than WA. Grey color represents the number of behavioral variables not showing significant difference in test accuracies between AA and WA. (B,C) For full-dataset models (when models were trained on the entire dataset), plot AA vs. WA accuracy difference (Pearson's correlation; vertical axis) against the difference in similarity between model-learned brain-behavior association patterns and true group-wise brain-behavior association patterns (horizontal axis). Each red dot represents a behavioral measure.
